# Advancements in buccal drug delivery: a mechanistic review of fast-disintegrating electrospun nanofiber mats

**DOI:** 10.1039/d6na00624h

**Published:** 2026-10-16

**Authors:** Razia Rehman, Hasham Shafi, Syed Naiem Raza, Reyaz Hassan Mir, Suriya Rehman, Touseef Amna, M. Shamshi Hassan, Sami-ullah Rather, Nisar Ahmad Khan, Faheem A. Sheikh

**Affiliations:** a Department of Pharmaceutical Sciences, University of Kashmir Hazratbal Srinagar-190006 Jammu and Kashmir India nakhan@uok.edu.in +91-9419017186; b Nanostructure and Biomimetic Lab, Department of Nanotechnology, University of Kashmir Hazratbal Srinagar-190006 Jammu and Kashmir India faheemnt@uok.edu.in +91-9797717235; c Department of Epidemic Diseases Research, Institute for Research and Medical Consultation (IRMC), Imam Abdulrahman bin Faisal University Dammam 34212 Saudi Arabia; d Center for Translational Science, Florida International University Port St. Lucie 34987 Florida USA; e Department of Biology, Faculty of Science, Al-Baha University Al-Baha 65799 Saudi Arabia; f Department of Chemistry, Faculty of Science, Al-Baha University Al-Baha 65799 Saudi Arabia; g Chemical & Materials Engineering Department, Faculty of Engineering, King Abdulaziz University Jeddah 21589 Saudi Arabia

## Abstract

Buccal drug delivery enables rapid absorption by bypassing gastrointestinal degradation and hepatic first-pass metabolism. However, conventional films, tablets, and patches suffer from slow hydration and salivary washout, which often delay drug absorption before it can permeate the mucosa. Fast-dissolving (FD) electrospun nanofiber mats overcome these limitations. These mats disintegrate in under 30 s and release ≥80–90% of the loaded drug within ∼60 s, driven by their nanoscale fibers (100–900 nm), their enormous surface area, their high porosity, and the formation of amorphous solid dispersions during rapid solvent evaporation. The article reviews material selection (hydrophilic polymers such as polyvinylpolypyrrolidone, poly(vinyl alcohol), and pullulan), electrospinning parameters, and architectural variations (blend, coaxial, and multilayer) that enable instantaneous capillary-driven wetting, volumetric hydration, and erosion-mediated burst release. Mechanistic insights are supported by Fick's first law, demonstrating that transient supersaturation and reduced diffusion path lengths generate 2- to 3-fold higher early-phase mucosal flux than in conventional systems. Comprehensive characterization approaches, representative drug examples, and practical barriers to translation have also been highlighted in this review. Fast-dissolving nanofiber mats address key limitations of buccal drug delivery, particularly the challenge of rapid onset. Remaining challenges in scalability, reproducibility, and regulatory standardization can be addressed by developing better *in vitro* and *in vivo* models and by drawing early clinical conclusions.

## Abstract 2

Buccal drug delivery enables rapid absorption by bypassing gastrointestinal degradation and hepatic first-pass metabolism (data extracted *via* Perplexity; figures compiled and annotated using Microsoft Paint).

## Graphical abstract

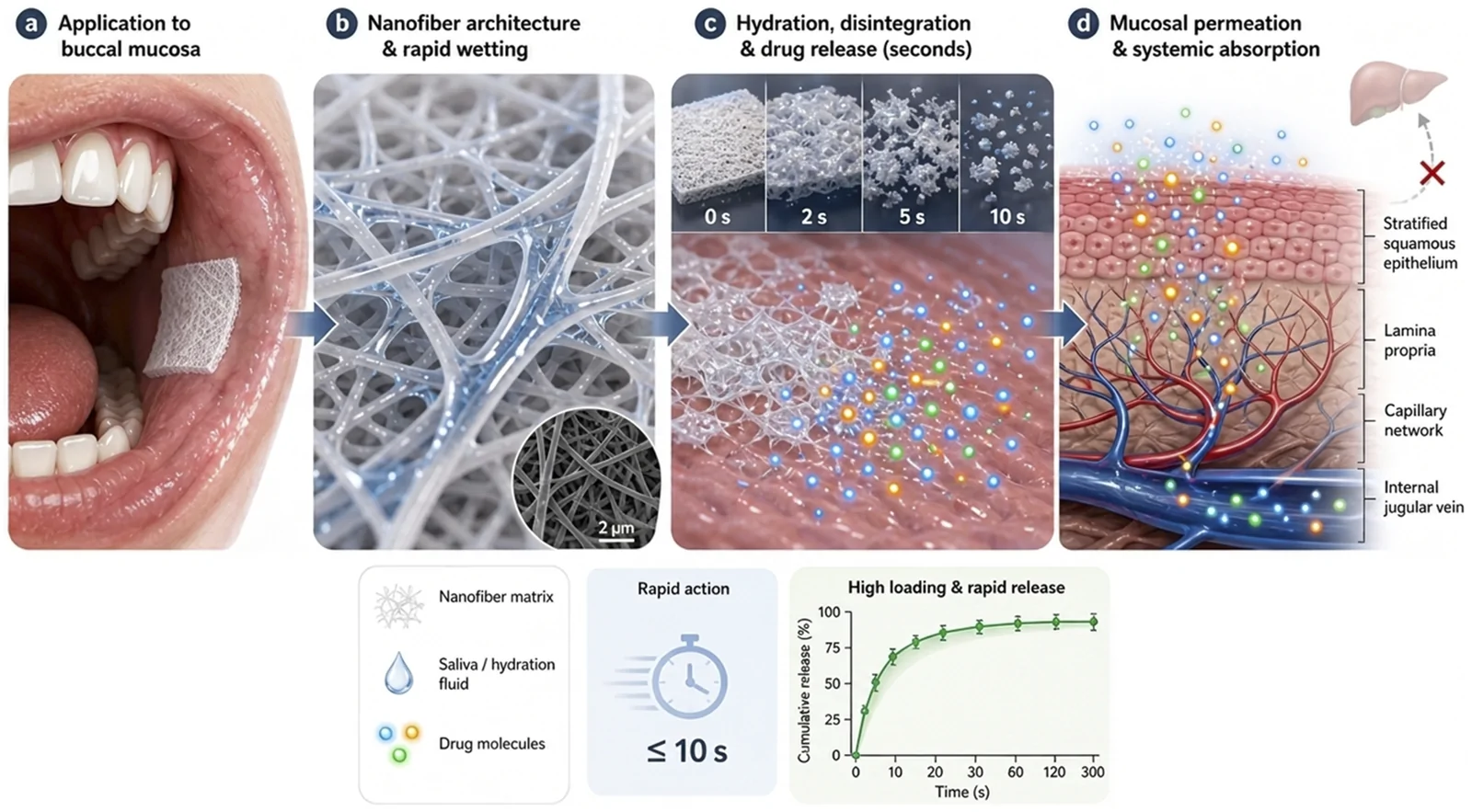

## 1. Literature search strategy

To provide a comprehensive and up-to-date overview of fast-disintegrating electrospun nanofiber mats for buccal drug delivery, a structured literature search was performed using PubMed, Scopus, Web of Science, and Google Scholar. Publications from 2000 to June 2026 were considered, with particular emphasis on studies published during the last five years to capture recent advances in electrospinning technologies, material engineering, and translational development. The search strategy employed combinations of keywords including electrospinning, electrospun nanofibers, fast-disintegrating nanofibers, buccal drug delivery, buccal films, oral mucosal delivery, amorphous solid dispersions, drug–polymer interactions, mucoadhesion, dissolution, permeation, and pharmaceutical manufacturing. The review primarily included peer-reviewed original research articles, review articles, and selected regulatory or pharmacopeial guidance documents directly relevant to electrospun buccal drug delivery systems. Studies focusing exclusively on non-pharmaceutical electrospinning applications, tissue engineering without drug delivery relevance, or unrelated oral dosage forms were excluded unless they provided mechanistic insights applicable to buccal nanofiber systems. Preference was given to studies offering detailed physicochemical characterization, mechanistic understanding, manufacturing considerations, and translational perspectives. Reference lists of key publications were also examined to identify additional relevant literature and ensure comprehensive coverage of the field.

## 2. Background and rationale for fast-dissolving (FD) buccal electrospun nanofiber (NF) mats

Buccal drug delivery has emerged as a valuable substitute to conventional oral administration of immediate-release drugs, enabling their rapid systemic absorption while circumventing gastrointestinal (GI) limitations and hepatic first-pass metabolism (FPM).^1,2^ The buccal mucosa, a highly vascularized and permeable tissue between the gums and the inner lining of the cheek, allows drugs to diffuse directly into systemic circulation *via* the internal jugular vein (Scheme 1a), thereby bypassing FPM and GI degradation.^1,3^ This route is particularly effective in delivering compounds with poor oral bioavailability, GI instability, and/or requirements for rapid therapeutic onset, such as antiemetics, anxiolytics, and analgesics.^4^ The stratified squamous epithelium (∼500–800 µm thick) of the buccal mucosa serves as the primary permeability barrier for drug absorption (Scheme 1b) and provides an efficient permeation interface, supporting rapid attainment of plasma concentrations through short diffusion distances and high vascularity.^5,6^ Patient compliance is enhanced by non-invasive self-administration, suitability for those with swallowing difficulties, and the ability to terminate dosing by manual removal.^7^ Continuous salivary flow (0.5–1.5 L per day; unstimulated ∼0.3–0.5 mL min^−1^)^8,9^ and rapid mucosal turnover further enable repeated use with low damage risk, eliminating GI transit delays.^9^

**Scheme 1:**
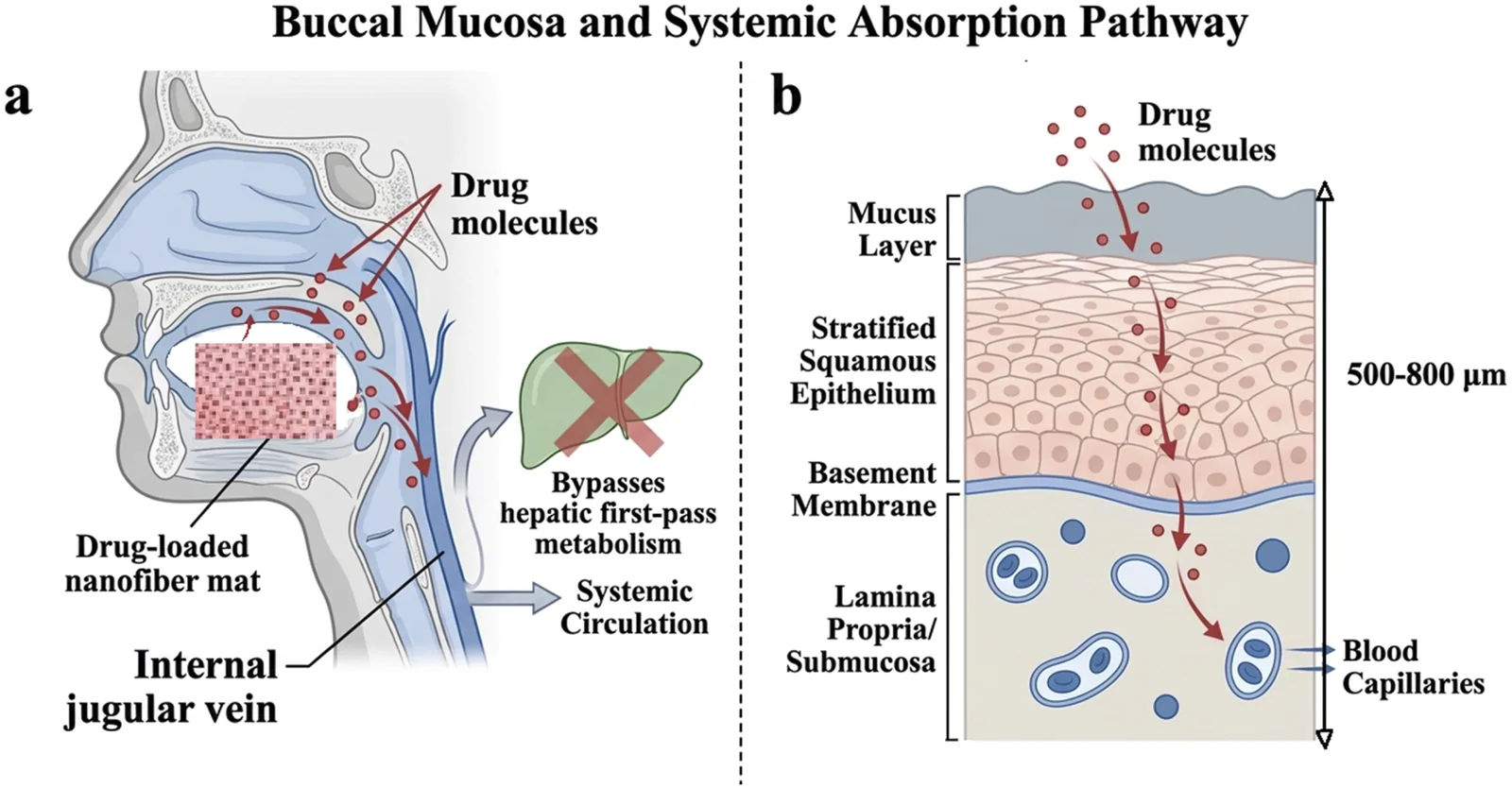
(a) Placement of a drug-loaded NF mat in the buccal cavity, showing hydration-driven release, mucosal permeation, and systemic uptake *via* the internal jugular vein, evading FPM. (b) Cross-section of the buccal mucosa, depicting diffusion across the mucus layer, epithelium, basement membrane, and into lamina propria capillaries (blue circles). Illustration fragments were generated with the assistance of Perplexity (AI-image generation) and manually refined and assembled using Paint software.

Despite these benefits, conventional buccal dosage forms, such as solvent-cast films, tablets, and patches, face constraints that hinder rapid dissolution and delayed therapeutic onset.^10^ These arise from slow hydration kinetics, salivary washout, and the structural barrier properties of the buccal epithelium.^11^ Conventional films and tablets rely primarily on polymer hydration and swelling before initial drug release begins.^12^ This process inherently delays disintegration and absorption, often resulting in wetting times of several minutes (Scheme 2a).^9^ For example, hydroxypropyl methylcellulose (HPMC)-based buccal films showed prolonged swelling indices and slow drug release, limiting their utility for rapid-onset therapy.^13^ Buccal patches, designed as non-dissolving matrix systems with backing layers and release-retardant polymers such as sodium carboxymethylcellulose (NaCMC) and HPMC, sustain drug release for 24 hours or more (Scheme 2b), which is unsuitable for fast buccal applications.^14^ Collectively, these yield diffusion-limited profiles mismatched with the buccal window. Beyond formulation constraints, physiological factors exacerbate these issues. For instance, continuous salivary flow causes rapid washout of non-adherent or partially hydrated forms, reducing residence time and drug absorption efficiency (Scheme 2c). This saliva washout effect can result in premature swallowing of the dosage before adequate permeation occurs.^15^ Even when residence is maintained, the lipid-rich buccal epithelium presents a substantial permeation barrier, restricting the flux of hydrophilic and/or high-molecular-weight compounds *via* tight junctions.^11,16^ Thus, conventional systems suffer from temporal misalignment, with slow liberation conflicting with brief exposure (∼60–120 s before clearance).^17^ Hence, the primary formulation challenge in fast-dissolving buccal systems is not long-term retention on the cheek mucosa but rather ensuring sufficiently rapid drug release and permeation kinetics to establish a high concentration gradient before salivary washout and swallowing occur.

**Scheme 2:**
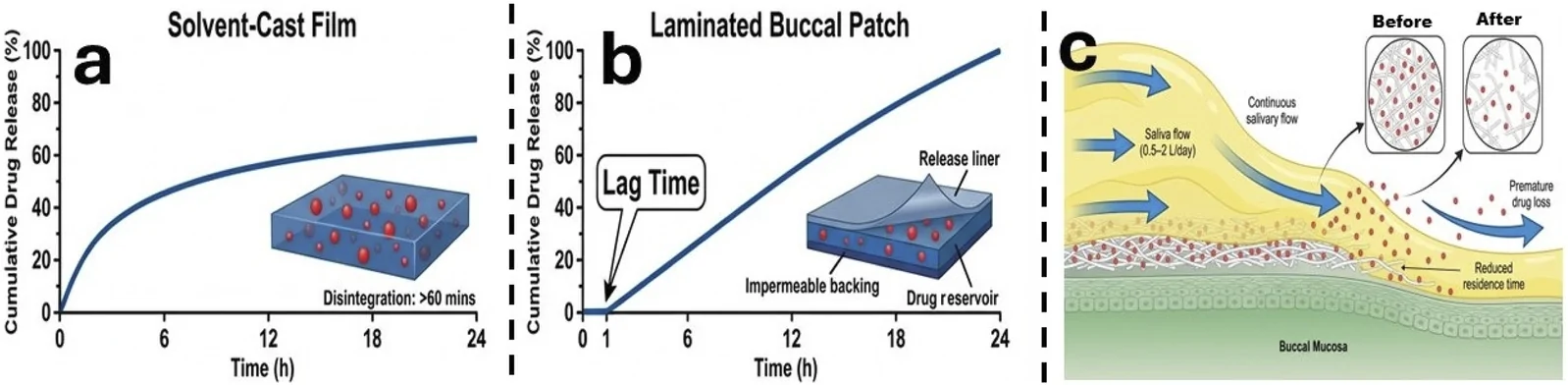
(a) Release profile of a conventional solvent-cast buccal film showing gradual drug release over hours due to slow matrix hydration and disintegration (>60 min). (b) Drug release from a laminated buccal patch illustrates an initial lag time caused by the impermeable backing layer and controlled diffusion from the drug reservoir through the release liner. (c) Salivary flow (0.5–1.5 L per day) diluting/removing the released drug; insets show before/after-exposure matrix disruption. Graphical components were created with Perplexity (AI-image generation) and further edited manually using Paint software.

In response to these limitations, electrospun NF mats have emerged as a structurally distinct platform capable of enabling true fast buccal drug delivery^18^ through nanoscale fibers (100–900 nm diameters) with exceptional surface-area-to-volume (SA/*V*) ratios and high porosity, enabling instantaneous wetting and absence of diffusional barriers.^19–21^ Additionally, rapid solvent evaporation during electrospinning frequently converts crystalline drugs into amorphous solid dispersions, thereby reducing free energy and enhancing solubility and dissolution rates.^22,23^ This architecture, characterized by interconnected pores and molecular dispersion, facilitates volumetric hydration, minimizing lag and synchronizing release with salivary dynamics (Scheme 3).^24^

**Scheme 3:**
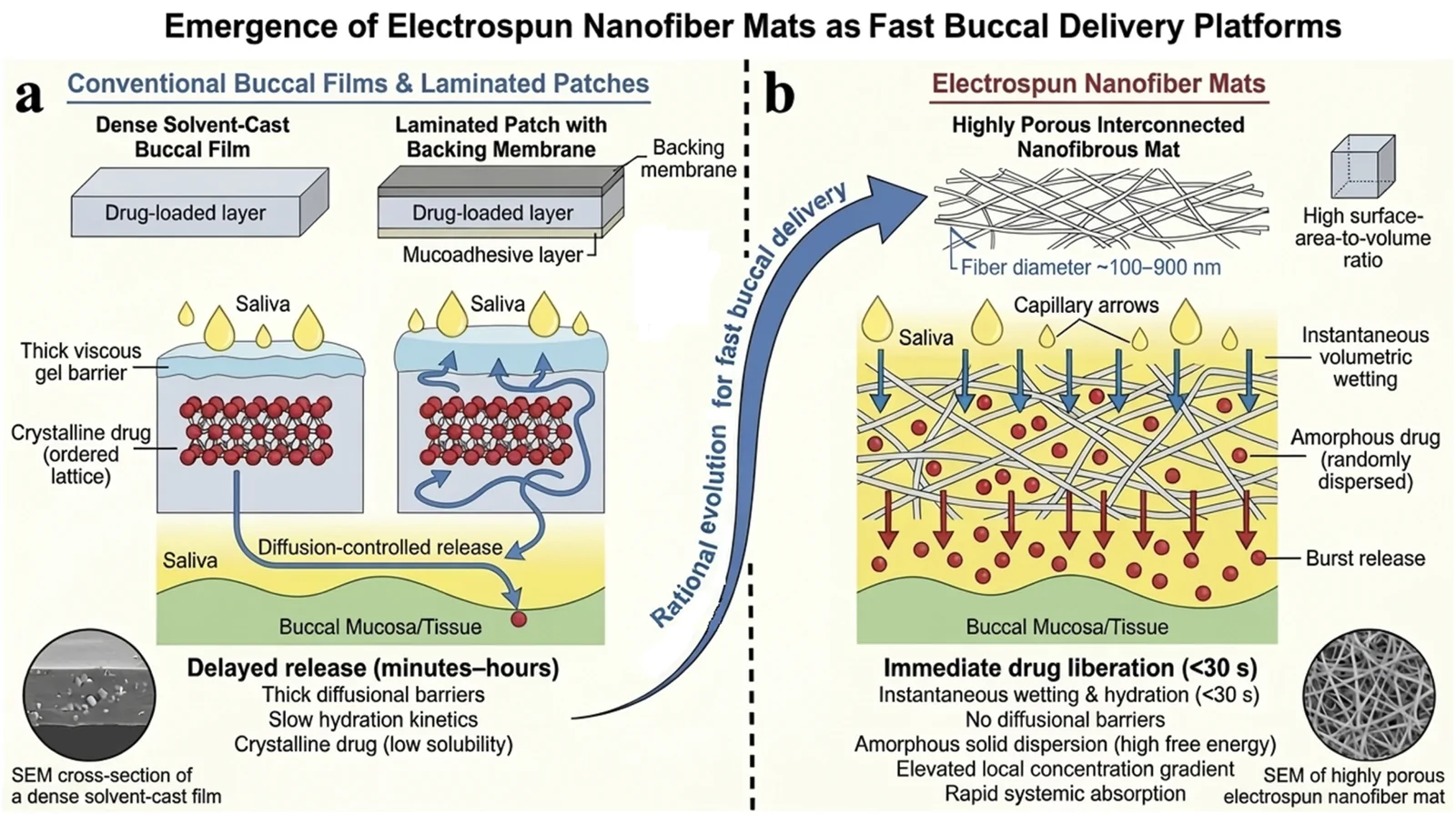
(a) Conventional buccal delivery systems, including solvent-cast films and laminated patches, typically possessing dense matrices with crystalline drug domains. Drug release occurs primarily through diffusion-controlled mechanisms, and hydration of the viscous gel barrier formed upon contact with saliva results in delayed release (minutes to hours). (b) Electrospun NF mats exhibiting a highly porous and interconnected fibrous architecture (fiber diameter ∼100–900 nm) with a high SA/*V* ratio. Rapid capillary-driven wetting and amorphous drug dispersion enable instantaneous hydration and burst drug release, facilitating rapid permeation across the buccal mucosa and faster systemic absorption. Illustration segments were developed using Perplexity (AI-image generation) and manually refined using Paint software.

FD buccal NF mats, a specialized subclass of transmucosal drug delivery systems, are fabricated *via* electrospinning. These are engineered to achieve rapid disintegration, immediate drug release, and rapid systemic exposure within the buccal cavity.^25,26^ Before proceeding further, it is necessary to define what “FD” actually means. Within the framework of this review, “fast-dissolving” buccal systems are defined as electrospun nanofibrous matrices engineered to undergo rapid structural disintegration (<30 seconds) and achieve substantial drug release (≥80–90% of the loaded dose) within approximately 60 seconds, accommodating physiological variability up to ∼120 seconds in the buccal exposure window, while mechanistically enhancing early-phase mucosal drug flux *via* immediate hydration, surface-mediated dissolution, and transient supersaturation at the buccal interface. This definition integrates both temporal and physiological criteria to reflect the limited mucosal exposure window characteristic of the buccal cavity. This threshold is not arbitrary; it is grounded in (1) empirical data repeatedly reported for electrospun NF mats, *e.g.*, Ponrasu *et al.* (60 s full dissolution),^20^ Alshaya *et al.* (≤12 s disintegration),^26^ Sharifi *et al.* (0.4 s disintegration, T90% in 1 min)^25^ and (2) the physiology of the non-mucoadhesive buccal environment,^24,27^ differentiating FD NFs from sustained patches (hours, diffusion-controlled)^28^ or FDOFs (5–30 s, bulk erosion) (Table 1). Unlike scaffolds for healing and/or prolonged local delivery, FD mats facilitate burst systemic exposure for acute needs and rationally evolve transmucosal platforms. Mechanistically, FD performance integrates nanoscale diameter (high SA/*V*), porosity (capillary infiltration), and amorphous states (elevated activity), yielding erosion-driven burst over swelling/diffusion. By concentrating release in the initial window, these mats optimize flux before washout, offering a distinct approach to oral drug delivery.^24^ A concise comparison of these distinctions across key parameters is provided in Table 1. It is important to note that, while the present review is focused on buccal delivery, a substantial portion of the foundational literature on FD electrospun NF mats originates from sublingual or orodispersible (oral) systems. The rationale is that the non-keratinized mucosa of the buccal and sublingual regions shares key physiological features: a thin stratified squamous epithelium (≈500–800 µm), high vascularity, similar salivary composition, and limited exposure windows that govern rapid hydration, disintegration, and transmucosal flux. Consequently, the mechanistic principles (capillary-driven wetting, volumetric hydration, amorphous solid dispersion, and burst release) established in sublingual/oral fast-dissolving NF studies provide mechanistic insights relevant to buccal applications. Where studies specifically report buccal or sublingual data, they are cited accordingly; otherwise, the cited oral fast-dissolving examples are included to illustrate the shared nanoscale architecture and performance attributes that are equally applicable in the buccal cavity. Overall, the evidence discussed in this review comprises three complementary categories: direct buccal studies, sublingual fast-dissolving nanofiber studies, and oral/orodispersible nanofiber studies used for mechanistic extrapolation. Direct buccal investigations provide the strongest evidence for mucoadhesion, permeation, and local physiological performance, whereas sublingual studies contribute clinically relevant information on rapid transmucosal absorption and early systemic exposure. Oral/orodispersible nanofiber studies are included primarily to illustrate shared nanoscale mechanisms such as capillary-driven wetting, amorphous stabilization, rapid disintegration, and burst drug release. Conclusions related specifically to buccal performance are therefore based preferentially on studies conducted in buccal or *ex vivo* buccal models, while extrapolated evidence is used to support mechanistic interpretation rather than to claim equivalent clinical outcomes. For clarity, representative examples throughout the review and in Table 3 are explicitly labeled as buccal, sublingual, or oral/orodispersible evidence, allowing readers to distinguish data derived directly from buccal studies from mechanistic evidence extrapolated from related mucosal delivery systems.

**Table 1:** Comparison of FD buccal NF mats with conventional oral/buccal dosage forms, highlighting structural and performance distinctions relevant to rapid systemic delivery

Parameter/system	FD buccal NF mats	Solvent-cast films	Mucoadhesive patches	FDOFs/orodispersible tablets	References
Architecture	Nanoscale fibrous network (high SA/*V*, porous)	Continuous planar film (low porosity)	Multilayer/backed with rate-control	Monolithic films/tablets (bulk matrix)	29 and 30
Disintegration time	<30 s (often ≤15 s; capillary collapse)	30–180 s (swelling-dependent)	Minimal (<60 s; designed for retention)	5–30 s (oral breakup; swallowing-focused)	31 and 32
Release time (≥80–90%)	≤60 s (burst *via* amorphization/supersaturation)	Minutes (erosion/diffusion)	Hours (matrix diffusion)	Minutes (hydration; GI absorption)	20 and 32
Drug state	Amorphous (PXRD/DSC-confirmed; high activity)	Often crystalline (unless dispersed)	Crystalline; diffusion-limited	Crystalline/partially amorphous	32 and 33
Hydration mechanism	Volumetric (porosity-driven)	Surface swelling/erosion	Layer-constrained	Bulk dissolution	30 and 31
Early flux	High (SA-mediated gradient)	Moderate (barriers delay)	Low (prolonged profile)	Low buccal; GI-dominant	26 and 29
Residence	Transient (<minutes; optional mild adhesion)	Moderate (some mucoadhesion)	Hours (adhesive design)	Minimal (rapid swallowing)	24 and 34
Best niche	Acute onset (*e.g.*, antiemetics, pain)	Moderate systemic/local	Sustained local/controlled	GI systemic *via* dissolution	26 and 31
Key constraint	Fragility *vs.* speed balance	Swelling *vs.* flexibility	Retention *vs.* comfort	Dissolution *vs.* taste-masking	35 and 36
Scale-up	Emerging (needleless viable)	Established (casting)	Scalable (lamination)	Mature (tableting)	35 and 37

## 3. Design and fabrication principles for FD buccal NF mats

Material selection and fabrication in FD buccal NF mats are tailored to deliver rapid hydration, amorphous drug stabilization, and transient mucosal contact within the ∼60 s exposure window. Polymers and excipients must exhibit high aqueous affinity, miscibility with drugs, and minimal crosslinking to enable capillary infiltration and erosion-driven burst release. In contrast, electrospinning parameters organize these into nanoscale architectures that amplify performance.^30^

### 3.1 Material characteristics for core functions

Polymers used in FD NF mats must exhibit high aqueous affinity and minimal resistance to solvent penetration, which aid in forming viscous gel barriers that could undesirably delay drug release. Hydrophilic polymers drive volumetric hydration (solvent uptake across the entire matrix rather than limited to the surface) and structural collapse, with low molecular weight and low degree of crystallinity accelerating faster dissolution kinetics.^38^ A recent buccal electrospun formulation exemplifies this material-driven behavior: polyvinylpyrrolidone-based nanofibrous mats incorporating sildenafil and glycyrrhizin disintegrated rapidly (≤4 ± 1 s) under buccal-relevant conditions. This was attributed to the non-crosslinked hydrophilic polymer matrix facilitating immediate aqueous penetration and rapid structural collapse. The observed edge-to-center disintegration pattern highlights how polymer solubility and nanofibrous architecture collectively govern matrix erosion, enabling swift conversion of the solid matrix into a drug-containing solution layer at the mucosal interface within the limited exposure window.^31^ Furthermore, rapid matrix collapse must be accompanied by accelerated drug dissolution to establish a high concentration gradient at the epithelial interface. This requires materials capable of stabilizing high-energy amorphous states and enhancing wettability. Beyond improving wettability, the ability of polymers to stabilize the amorphous drug state is governed by molecular-level intermolecular interactions.^39,40^ For instance, hydrogen bonding, dipole–dipole interactions, and van der Waals forces between drug molecules and polymer functional groups reduce molecular mobility and inhibit drug–drug association during and after electrospinning.^23^ Polymers such as polyvinylpyrrolidone (PVP), poly(vinyl alcohol) (PVA), hydroxypropyl methylcellulose (HPMC), and pullulan contain hydrogen-bond accepting or donating groups that interact with complementary functionalities present in many pharmaceutical compounds, thereby preventing molecular rearrangement into energetically favorable crystalline lattices.^41,42^ From a thermodynamic perspective, drug–polymer miscibility is frequently evaluated using the Flory–Huggins interaction parameter (*χ*), where lower *χ* values indicate greater thermodynamic compatibility between the drug and polymer, reducing the driving force for phase separation and subsequent recrystallization.^43,44^ In addition, polymers possessing relatively high glass-transition temperatures decrease molecular mobility within the electrospun matrix, kinetically stabilizing supersaturated amorphous dispersions throughout storage.^45^ Consequently, polymer selection in FD NFs should be guided not only by hydrophilicity but also by thermodynamic compatibility, intermolecular interaction potential, and glass-transition behavior to preserve the amorphous state throughout manufacturing and storage while sustaining rapid dissolution.^46^ Because amorphous drugs generally possess higher Gibbs free energy and thermodynamic activity than their crystalline counterparts, they can exhibit enhanced apparent solubility and dissolution rates upon hydration.^22^ PVA's miscibility facilitates this during spinning, as confirmed by XRD/DSC in ondansetron-loaded PVA NFs (complete amorphization, ∼10 s disintegration, >90% release in 90 s).^47^ In another example, gelatin–caffeine mats similarly trapped caffeine amorphously, yielding <30 s dissolution despite its crystallization propensity.^48^ Though FD systems prioritize rapid dissolution, a minimal degree of mucosal interaction may enhance effective absorption within the short exposure window. Materials contributing to transient mucoadhesion can reduce premature salivary washout without compromising matrix disintegration.^34^ Polymers containing cationic or hydrogen-bonding functional groups, such as chitosan and related derivatives, can interact with mucin glycoproteins through electrostatic and intermolecular forces.^49^ However, in FD NF systems, mucoadhesion must remain moderate and short-lived. Excessive crosslinking or high-viscosity gel formation may impede hydration and undermine the objective of rapid dissolution. Therefore, the material design challenge lies in achieving an equilibrium between immediate dissolution and sufficient early-phase epithelial contact. For instance, cationic chitosan in pullulan blends provides short-lived mucoadhesion *via* hydrogen bonding (rapid <60 s release with measurable epithelial contact).^50^

Certain FD systems reduce or eliminate conventional polymer backbones, relying on highly water-soluble excipients or self-assembled inclusion complexes to form electrospinnable matrices.^51^ These minimal-matrix approaches reduce structural barriers to dissolution and can achieve extremely rapid disintegration upon contact with saliva.^52^ While such systems may offer exceptional dissolution kinetics, considerations of mechanical robustness, handling stability, and dose-loading capacity become increasingly important.^53^ Thus, material simplicity must be balanced against practical functionality. Cyclodextrin inclusion complexes extend this to poorly soluble drugs; hydroxypropyl-β-cyclodextrin/ibuprofen NFs, which are polymer-free, dissolve near-instantaneously due to their intrinsic solubility and the absence of matrix barriers.^54^ Overall, FD buccal NF systems are not defined by a specific polymer class but by coordinated material design aligned with temporal constraints. Therefore, achieving substantial drug release within ≈60 s from these polymers requires:

• High aqueous affinity and rapid solvent penetration.

• Favorable drug–polymer miscibility to sustain amorphous states.

• Minimal crosslink density and absence of diffusion-limiting gel layers.

• Fiber diameters and porosity supporting capillary-driven hydration.

• Transient mucosal interaction without prolonged structural integrity.

### 3.2 Fabrication principles and architectural designs

Electrospinning converts materials into FD systems by enabling simultaneous control over fiber diameter, interfiber porosity, and solid-state drug dispersion in a single processing step.^30^ Thinner diameters (100–300 nm) *via* high voltage (15–20 kV) and low flow rates (0.05 mL min^−1^) maximize SA/*V* ratios for instantaneous wetting, as in low-molecular-weight PVA donepezil webs (9–13 s tongue disintegration, outperforming cast films) (Fig. 1a).^55^ Porosity determines whether hydration proceeds as a surface phenomenon or a volumetric event. Electrospinning inherently generates interconnected fibrous networks that promote capillary-driven saliva infiltration. Lower polymer concentrations, appropriate environmental humidity, and controlled deposition density enhance interfiber spacing, thereby enabling rapid internal wetting and preventing diffusion-limiting gel layers.^30^ Dense packing or excessive fiber fusion shifts the system toward film-like behavior, undermining the objective of rapid disintegration. Porosity, tuned by low polymer concentration and humidity, ensures interfiber spacing for bulk infiltration: PVP-ketoprofen mats retained ∼0.5 s of blank wetting post-scale-up, though high loading (>20 wt%) densified networks, extending times to >60 s (Fig. 1b).^35^

**Fig. 1:**
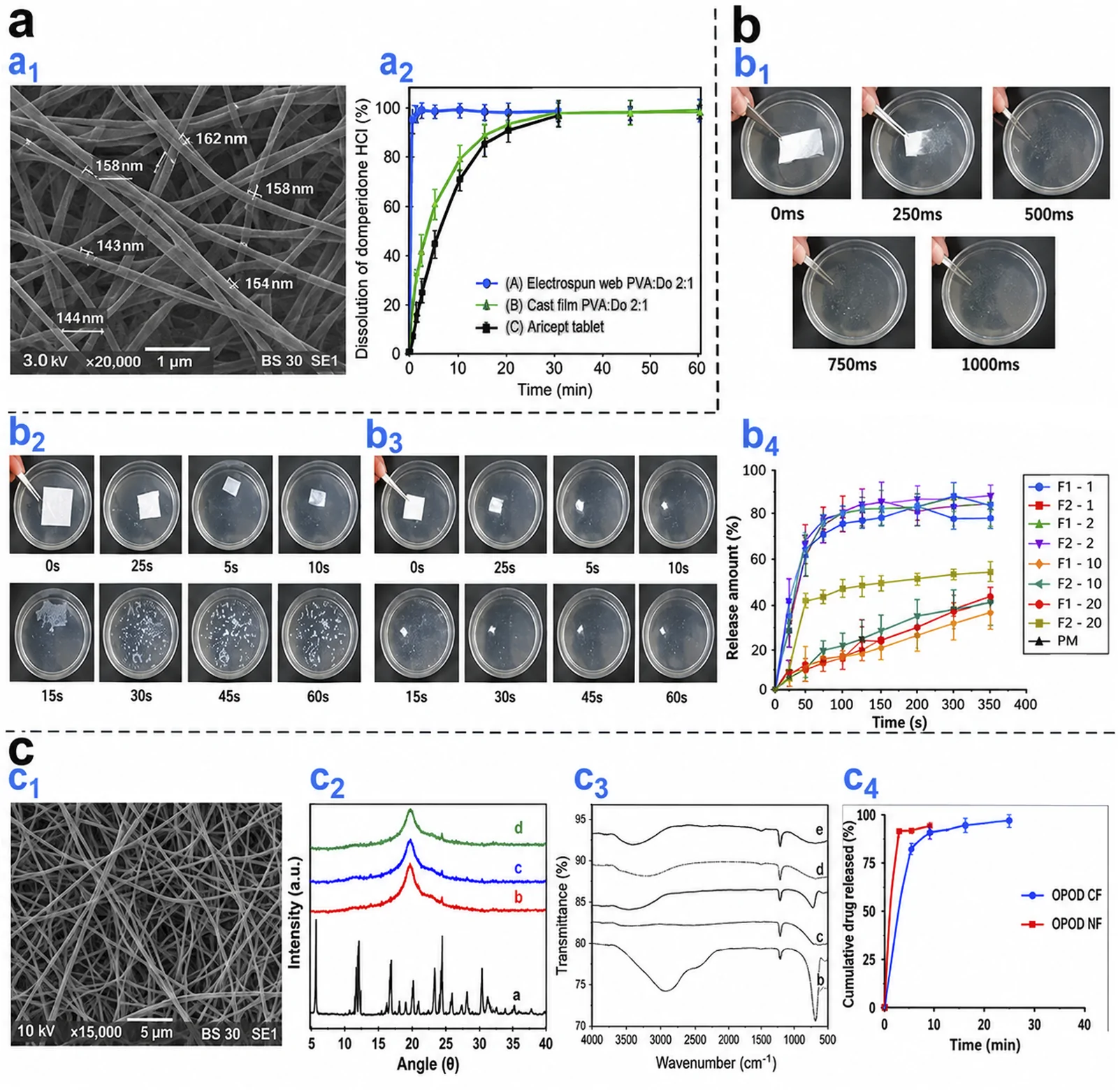
Process-driven structural determinants in FD buccal NF fabrication, illustrating fiber diameter, porosity, and solid-state transformation. (a) Scanning Electron Microscopy (SEM) image of low-molecular-weight PVA NFs (100–300 nm diameter) showing bead-free morphology and high surface area for 9–13 s disintegration (a_1_) and drug release profiles of PVA NFs compared to cast films and commercial tablets (a_2_).^55^ (b) PVP-based ketoprofen NF mats showing rapid wetting [∼0.5 s – blank (b_1_), <60 s – 9.09% w/w ketoprofen loaded (b_3_), >60 s – 23.08% w/w ketoprofen loaded (b_2_)]. *In vitro* dissolution profiles of electrospun NFs of F1 (9.09% w/w ketoprofen loaded), F2 (23.08% w/w ketoprofen loaded) and the physical mixture with different spinning flow rates (b_4_).^35^ (c) SEM image of PVA-ondansetron NFs confirming uniform bead-free fibers (c_1_), PXRD and DSC patterns of drug-loaded NFs confirming complete amorphization within the matrix (c_2_ and c_3_) for ∼10 s disintegration and release within 90 s (c_4_).^47^ The image fragments were adapted from different research papers: (a) adapted from ref. 55 under a Creative Commons Attribution 4.0 International License (CC BY-NC-ND 4.0), copyright 2010; (b) adapted from ref. 35 with permission from Elsevier, copyright 2021; and (c) adapted from ref. 47 with permission from Elsevier, copyright 2016.

Controlling fiber diameter and porosity, electrospinning process parameters govern the molecular organization of the drug within the nanofibers. These process parameters can therefore determine the extent of drug amorphization achieved during fiber formation.^56^ The formation of amorphous solid dispersions relies on ultrafast solvent removal, which kinetically immobilizes drug molecules before they can reorganize into energetically favourable crystalline lattices.^57^ In FD buccal systems, kinetic trapping is particularly advantageous because dissolution must occur before significant molecular reorganization or recrystallization can occur.^23^ This phenomenon is strongly influenced by applied voltage (electric field strength), solution viscosity, electrical conductivity, surface tension, and solvent volatility. Adequate viscosity promotes sufficient polymer chain entanglement to produce stable, bead-free fibers while simultaneously restricting molecular diffusion that could otherwise facilitate crystal nucleation. Conversely, solutions with inadequate viscosity generate unstable jets and drug-rich domains that increase the likelihood of phase separation.^58^ Furthermore, electrical conductivity determines the charge density carried by the jet and consequently its influence on elongational stretching. Increased stretching accelerates solvent evaporation and molecular dispersion, thereby reducing local supersaturation gradients that favor crystallization.^59^ Surface tension governs Taylor cone stability and jet breakup; excessive surface tension promotes bead formation and heterogeneous drug distribution, whereas balanced surface tension supports continuous fiber formation with homogeneous molecular encapsulation.^60^ Likewise, highly volatile solvent systems promote rapid vitrification of the polymer matrix, trapping drug molecules in a metastable amorphous state before sufficient molecular mobility exists for crystal growth. During this rapid solidification process, polymer chains become preferentially oriented along the fiber axis. Concurrently, drug molecules remain molecularly dispersed within the polymer network through hydrogen bonding and other intermolecular interactions.^61^ Jointly, these physicochemical and processing parameters transform electrospinning from a simple fiber fabrication technique into a highly effective amorphization process that suppresses nanostructure crystallization while simultaneously generating the structural characteristics responsible for rapid dissolution. Uniform dispersion minimizes localized drug aggregation and reduces variability in dissolution onset. Importantly, the fabrication process does not merely carry the drug; it kinetically defines its energetic state at the point of administration.^62,63^ The kinetic entrapment of amorphous forms of the drug *via* rapid evaporation is evident from ondansetron NFs (326 nm fibers, no PXRD and DSC peaks, 90 s full release) (Fig. 1c).^47^

Blend electrospinning (Scheme 4a) is the most common method for fabricating NFs with burst-type release, provided that drug–polymer miscibility is adequate.^64^ For instance, poly(ethylene oxide) (PEO)-based NFs incorporate potassium iodate at loadings of up to 21 wt%. Despite the relatively high loading, optimized formulations maintained smooth morphology (Fig. 2a_1_) and exhibited rapid wetting (<20 s) and complete dissolution within 30–60 s (Fig. 2a_3_) under saliva-mimicking conditions. Increasing salt content beyond optimal thresholds induced bead formation (Fig. 2a_2_), illustrating how an excessive solid fraction can disrupt jet stability and compromise architectural uniformity.^65^

**Scheme 4:**
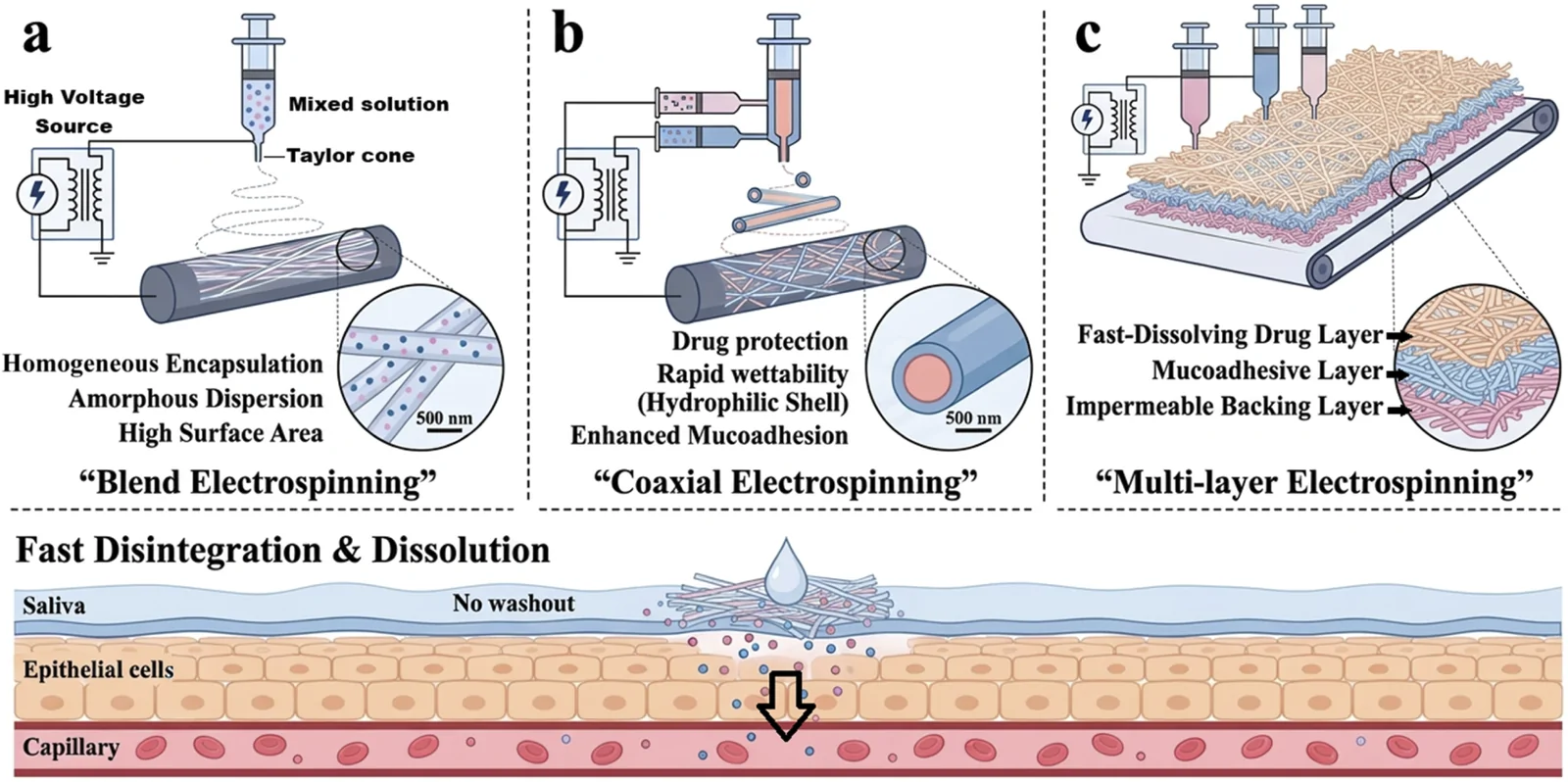
(a) Blend electrospinning: a drug–polymer solution is electrospun through a single spinneret under a high-voltage field, forming NFs with homogeneous drug encapsulation and amorphous dispersion within a high-surface-area fibrous network. (b) Coaxial electrospinning: core–shell NFs are produced using concentric needles, where the drug-containing core is surrounded by a hydrophilic polymer shell, enabling drug protection, rapid wettability, and enhanced mucoadhesion. (c) Multilayer electrospinning: sequential deposition of distinct fiber layers generates a modular architecture comprising a fast-dissolving drug layer, a mucoadhesive intermediate layer, and an impermeable backing layer, enabling unidirectional drug release and tunable dual-release profiles. The electrospun mats rapidly disintegrate upon contact with saliva, allowing immediate drug liberation and permeation across the non-keratinized buccal epithelium while minimizing salivary washout. Illustration segments were developed using Perplexity (AI-image generation) followed by manual refinement using Paint software.

**Fig. 2:**
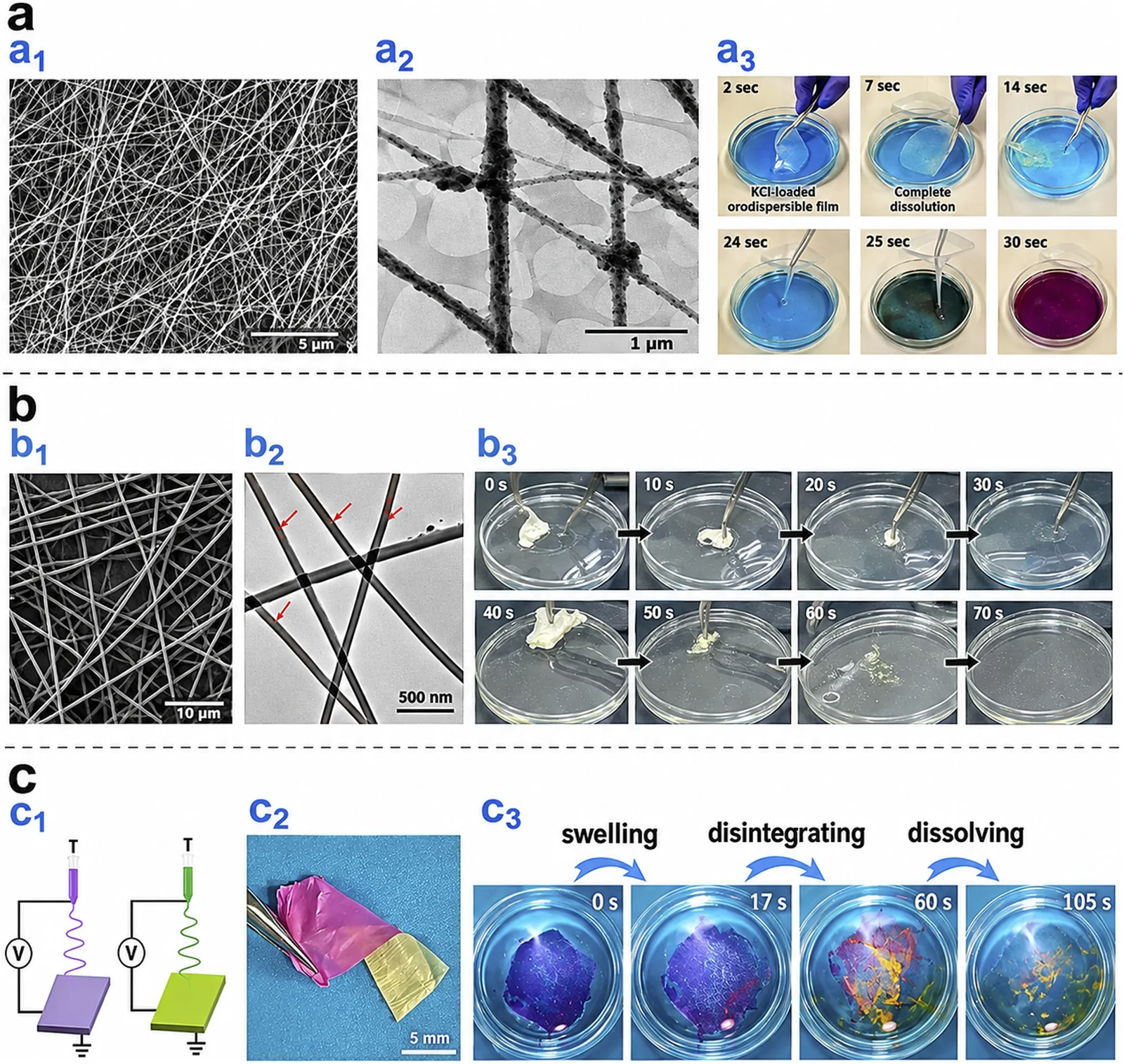
Architectural variations in FD buccal NF fabrication and their impact on rapid release. (a) SEM of blend electrospun PEO-based NFs with potassium iodate (KIO_3_) (21 w/w%) (a_1_), showing homogeneous dispersion and time-lapse dissolution (<20 s wetting, 30 s complete) (a_3_); TEM image of PEO NFs loaded with 43% KIO_3_, clearly showing beaded morphology and formation of nanocrystals at higher loading concentration (a_2_).^65^ (b) SEM of coaxial PVP-based NFs with a nifedipine/atorvastatin core–shell structure (∼968 nm diameter), confirming no drug crystals on the surface, indicating the successful integration of this drug into the fibers (b_1_); TEM illustrating two distinct layers, representing the inner and outer layers of the core–shell nanofibrous system (b_2_); disintegration test showing the disintegration time for blank nanofibers (≤2 s) and the drug-loaded nanofibers (≤12 s), respectively (b_3_).^26^ (c) Schematic illustration of the sequential electrospinning approach used to fabricate the dual drug-loaded bilayered gelatin nanofibrous membrane (∼88 µm thickness) (c_1_), digital image of the obtained dual drug-loaded nanofibrous membrane (c_2_), fast-dissolving process of the dual drug-loaded bilayered gelatin nanofibrous membrane (c_3_).^66^ The image fragments were adapted from different research papers: (a) adapted from ref. 65 with permission from American Chemical Society, copyright 2015; (b) adapted from ref. 26 under a Creative Commons Attribution 4.0 International License (CC BY), copyright 2022; and (c) adapted from ref. 66 under a Creative Commons Attribution 4.0 International License (CC BY), copyright 2022.

Beyond single-fluid blend electrospinning, architectural variations provide additional control over release behavior and mucosal interaction while retaining FD performance when shells and layers are kept thin and hydrophilic.^50^ Coaxial electrospinning produces core–shell fibers (Scheme 4b), enabling spatial separation of drug and functional excipients.^67^ In FD systems, this strategy can protect labile drugs while allowing the shell layer to modulate wetting or transient mucoadhesion.^63^ The feasibility of maintaining high performance in a core–shell configuration has been demonstrated using PVP-based coaxial NFs that incorporate two poorly soluble drugs in spatially distinct compartments. PVP-nifedipine/atorvastatin mats (∼968 nm) (Fig. 2b_1_) confirmed distinct layers *via* TEM (Fig. 2b_2_) with 12 s disintegration (despite architectural layering) (Fig. 2b_3_) and rapid dual release, preserving hydration *via* thin, wettable shells.^26^ Liu *et al.* further demonstrate coaxial feasibility for fast oral delivery by converting unspinnable licorice fluid extracts into core–shell NFs (PVP/sucralose shell) with complete dissolution in seconds while protecting core actives.^68^ Sequential or multi-fluid electrospinning enables formation of layered mats, combining rapidly dissolving layers with transient mucoadhesive or backing components (Scheme 4c).^69^ Such configurations can reduce premature salivary washout while preserving rapid drug liberation from the primary dissolving layer.^70^ However, increased structural thickness or incorporation of slowly hydrating backing layers may extend disintegration time beyond the desired <60 s window. The compatibility of multilayer architectures with rapid-dissolution objectives has been demonstrated using sequentially electrospun gelatin nanofibrous membranes designed to separate hydrophilic and hydrophobic drugs (Fig. 2c_1_) spatially. Gelatin bilayers (∼88 µm) separated hydrophilic/hydrophobic drugs (Fig. 2c_2_), achieving ∼105 s full collapse and simultaneous release through soluble layers (Fig. 2c_3_). These suit FD when total mass remains low and porosity high, avoiding diffusion lags.^66^ In summary, FD design converges materials and fabrication to embed rapid kinetics into architecture: hydrophilic matrices for function, spinning parameters for nanoscale precision, and variations for targeted modulation, ensuring burst performance without sustained-release trade-offs.

## 4. Mechanism of rapid drug release and mucosal permeation

FD buccal electrospun NF mats enable rapid systemic uptake *via* a coordinated cascade of events upon salivary contact: (i) instant wetting and capillary infiltration, (ii) fiber plasticization and disintegration, (iii) polymer dissolution with amorphous drug liberation, (iv) diffusion across the saliva layer, and (v) early-phase transmucosal permeation. This sequence, illustrated in Scheme 5(a-f), leverages nanoscale architecture for burst kinetics synchronized to the ∼60 s exposure window.^71^ Placement on the mucosa triggers immediate saliva absorption *via* capillary forces in the >80% porous network, driven by submicron fibers (100–900 nm) and high SA/*V* ratios.^19^ Hydrophilic polymers (*e.g.*, PVA or PVP) ensure instant wetting without surface lags seen in dense films, enabling volumetric hydration throughout the scaffold.^71^ Water plasticizes polymer chains, shifting from a glassy to a rubbery state and relaxing entanglements; disintegration follows rapidly as fiber junctions weaken and the matrix fragments into solvated pieces.^72^ This exposes surface-localized drugs early, initiating burst release (Scheme 5a–c).

**Scheme 5:**
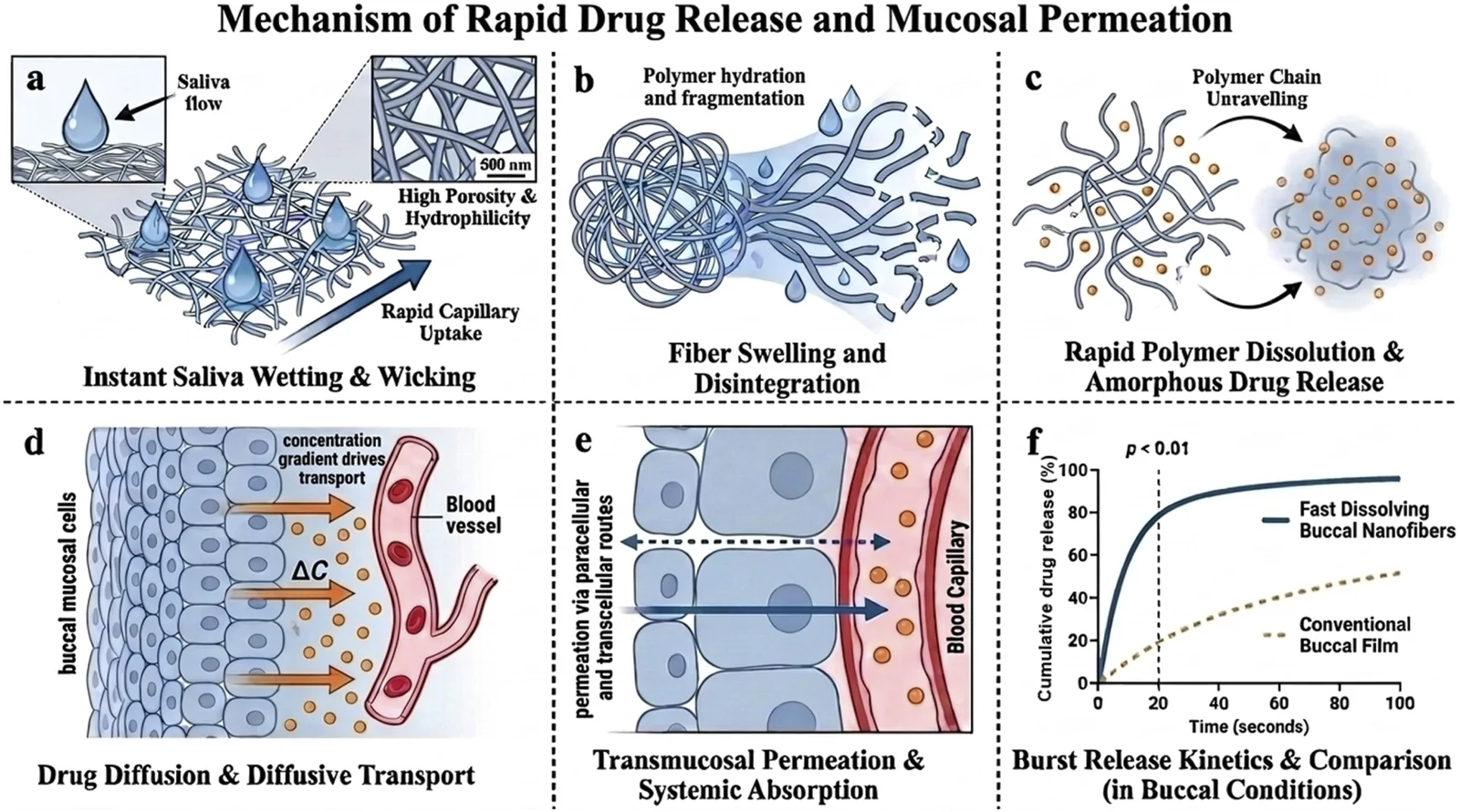
Step-by-step mechanistic cascade of FD drug release and permeation from electrospun NF mats in the buccal cavity. (a–c) Instant saliva wetting, capillary infiltration, fiber plasticization, and structural disintegration. (d) Rapid polymer dissolution, with the liberation of an amorphous drug, generates transient supersaturation at the mucosal interface and allows the solubilized drug to diffuse across the thin saliva layer. (e) Early-phase transmucosal permeation into the lamina propria capillaries and systemic circulation *via* the internal jugular vein. (f) Schematic comparison of dissolution profiles showing burst release from the FD NF mat *versus* the slow, diffusion-controlled release from a conventional solvent-cast film, all completed within the physiological ∼60 s exposure window before salivary washout. Illustration fragments were generated with Perplexity (AI-image generation) and subsequently refined manually in Paint.

Hydration rapidly dissolves the polymer matrix, liberating the drug in its amorphous form. For compounds that form stable amorphous dispersions (typically low-to-moderate dose, poorly water-soluble drugs), this creates transient supersaturation at the mucosal surface and a steep concentration gradient (Δ*C*) that drives high mucosal flux (Scheme 5d). The instantaneous flux is described by Fick's first law of diffusion:^19^
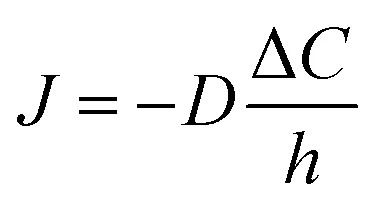
where *J* is flux, *D* the diffusion coefficient, Δ*C* the concentration gradient, and *h* the diffusion path length. Although the overall process is transient and better described by Fick's second law, the first-law approximation clearly shows how supersaturation and the absence of a gel layer boost early-phase permeation. The dissolution step itself follows Noyes–Whitney kinetics, further accelerated by the nanofibers' enormous surface area. In *ex vivo* studies, these mats consistently achieve mucosal flux 2–3 times higher than that of conventional films.^25^ The freed drug then diffuses across the ∼500–800 µm epithelium *via* trans- and paracellular routes (Scheme 5e), enhanced by intimate nanofiber-mucosa contact and optional tight-junction modulators such as chitosan.^30^ Because the entire payload is released before salivary clearance, efficient uptake occurs within the brief exposure window, accelerating *T*_max_ for acute therapies (Scheme 5f).^71^

### 4.1 Mechanistic validation from representative FD buccal NF systems

The cascade described above is experimentally validated in multiple FD buccal NF systems. For instance, Sharifi *et al.*'s PVP-diclofenac mats (387 nm fibers; 5% superdisintegrant) (Fig. 3a_1_) showed 0.4 s disintegration *via* capillary ingress (Fig. 3a_2_), full amorphization (XRD) (Fig. 3a_3_), and 120 s dissolution (Fig. 3a_4_), yielding 3.7 times *ex vivo* flux *vs.* films, confirming porosity-thermodynamics synergy.^25^ Similarly, Li *et al.*'s PVA-caffeine/riboflavin NFs (260–370 nm; ∼4.5 s wetting) (Fig. 3b_1_, b_2_ and b_4_) trapped drugs amorphously (no XRD peaks) (Fig. 3b_3_), achieving 100% release in <60 s with anomalous transport (Korsmeyer–Peppas *n* = 0.62–0.79; *R*^2^ > 0.95) (Fig. 3b_5_) and highlighting reduced paths and architecture-driven burst.^73^ These studies confirm that the combination of porosity, volumetric hydration, and amorphous solid dispersion drives erosion-mediated burst release independent of the drug's inherent solubility.

**Fig. 3:**
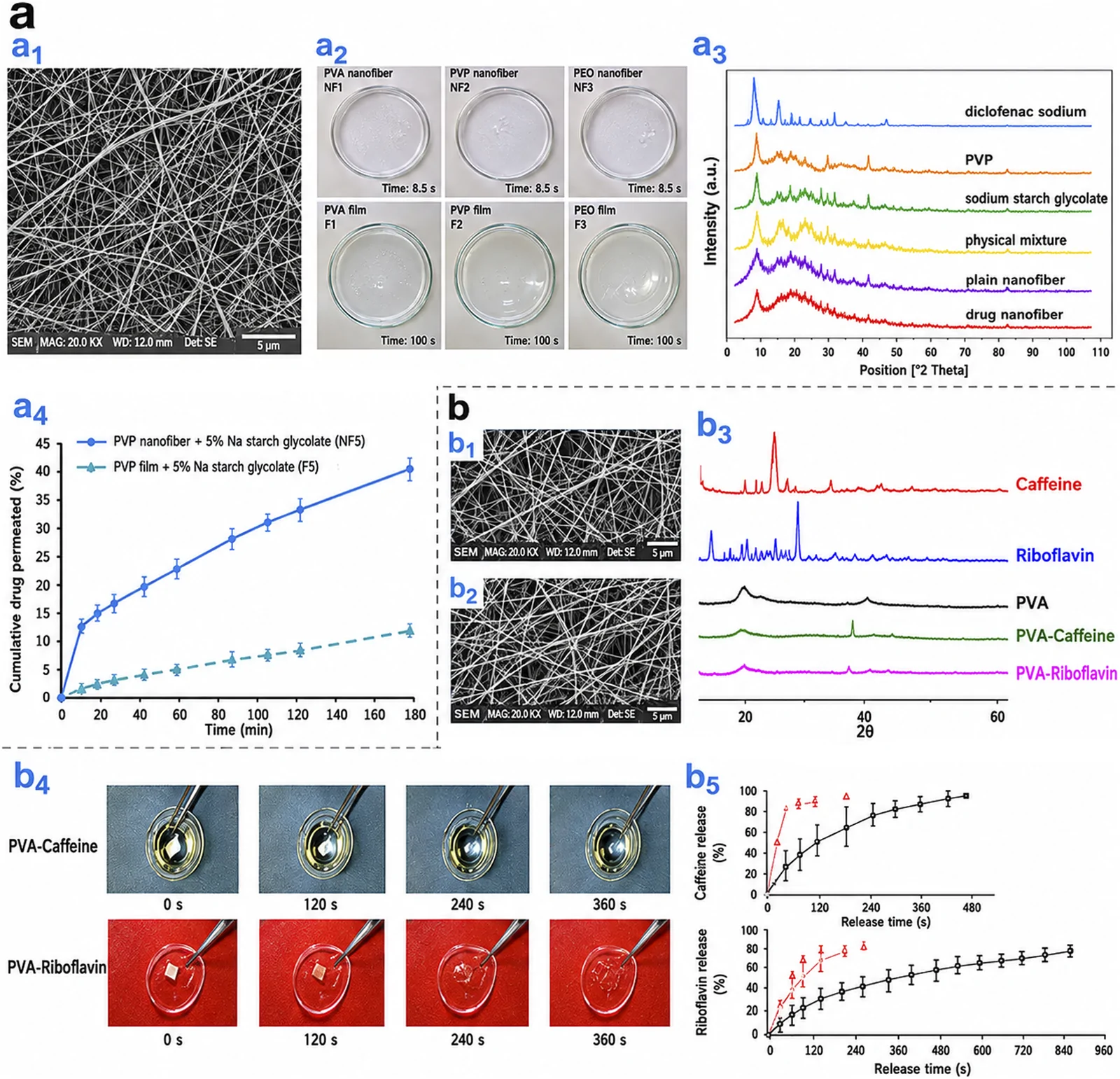
Mechanistic validation of rapid release in FD buccal NF mats. (a) SEM micrograph of diclofenac-loaded PVA NFs showing non-beaded morphology (∼380 nm) (a_1_), time-lapse disintegration of PVA NF showing 0.4 s disintegration and complete release within 60 s, illustrating capillary hydration and structural collapse (a_2_), XRD showing decreased intensity of drug peaks in an NF sample, confirming amorphization of the drug (a_3_), *ex vivo* permeation kinetics of 5% SSG PVP NFcompared to the cast film across the buccal mucosa, showing 3.7-fold higher permeation (a_4_).^25^ (b) SEM images of PVA/caffeine (b_1_) and PVA/riboflavin NFs (b_2_), revealing uniform fibers of 260–370 nm diameter without any formation of beads, XRD peaks showing complete absence of characteristic peaks of caffeine or riboflavin in an NF sample, implying amorphous conversion (b_3_), the disintegration time was about 1.5 s for both caffeine- and riboflavin-PVA NFs, with the riboflavin sample disintegrating into minute pieces that spread throughout the medium (b_4_), *in vitro* release profile of caffeine/riboflavin-loaded PVA NFs exhibiting 100% burst within 60 s, dotted lines show the fitting using Korsmeyer–Peppas' model for kinetic release (b_5_).^73^ The image fragments were adapted from different research papers: (a) adapted from ref. 25 with permission from Elsevier, copyright 2022; and (b) adapted from ref. 73 with permission from Elsevier, copyright 2013.

## 5. Characterization approaches

Characterization of FD buccal NF mats must causally link nanoscale architecture, drug dispersion, and hydration dynamics to sub-minute performance, thereby validating the translation of fabrication to a rapid release profile under dynamic buccal conditions (limited saliva, ∼60 s exposure window).^74,75^ Beyond routine analytics, methods prioritize morphology for wetting, solid-state properties for amorphization, disintegration kinetics, mechanical integrity, and dissolution profiles, mucoadhesion for retention, and long-term stability for shelf-life, ensuring FD traits endure without gel barrier formation or recrystallization.^76^

### 5.1 Morphological and topographical characterization

SEM quantifies fiber diameter (target <500 nm for high SA/*V*), bead defects, fiber connectivity, and porosity (>70–80% for capillary infiltration), directly tying to wetting speed.^77^ Atomic force microscopy (AFM) provides roughness/topography insights, critical for saliva spreading, particularly for fibers <200 nm in diameter.^64^ In domperidone PVA NFs, SEM showed bead-free 280–565 nm diameters (Fig. 4a) with open networks, enabling 4–8 s disintegration;^72^ pullulan/xanthan vitamin C NFs (181–261 nm) (Fig. 4b) confirmed >80% porosity for <3 s dissolution.^77^ Multilayer ciprofloxacin patches revealed interconnected layers (Fig. 4c_1_–c_3_) for burst antibiotic release.^78^ AFM on ondansetron PVA mats indicated low-roughness surfaces (Fig. 4d) for uniform <10 s hydration.^47^ These verify that the architecture supports volumetric destabilization over surface-limited swelling.^79^

**Fig. 4:**
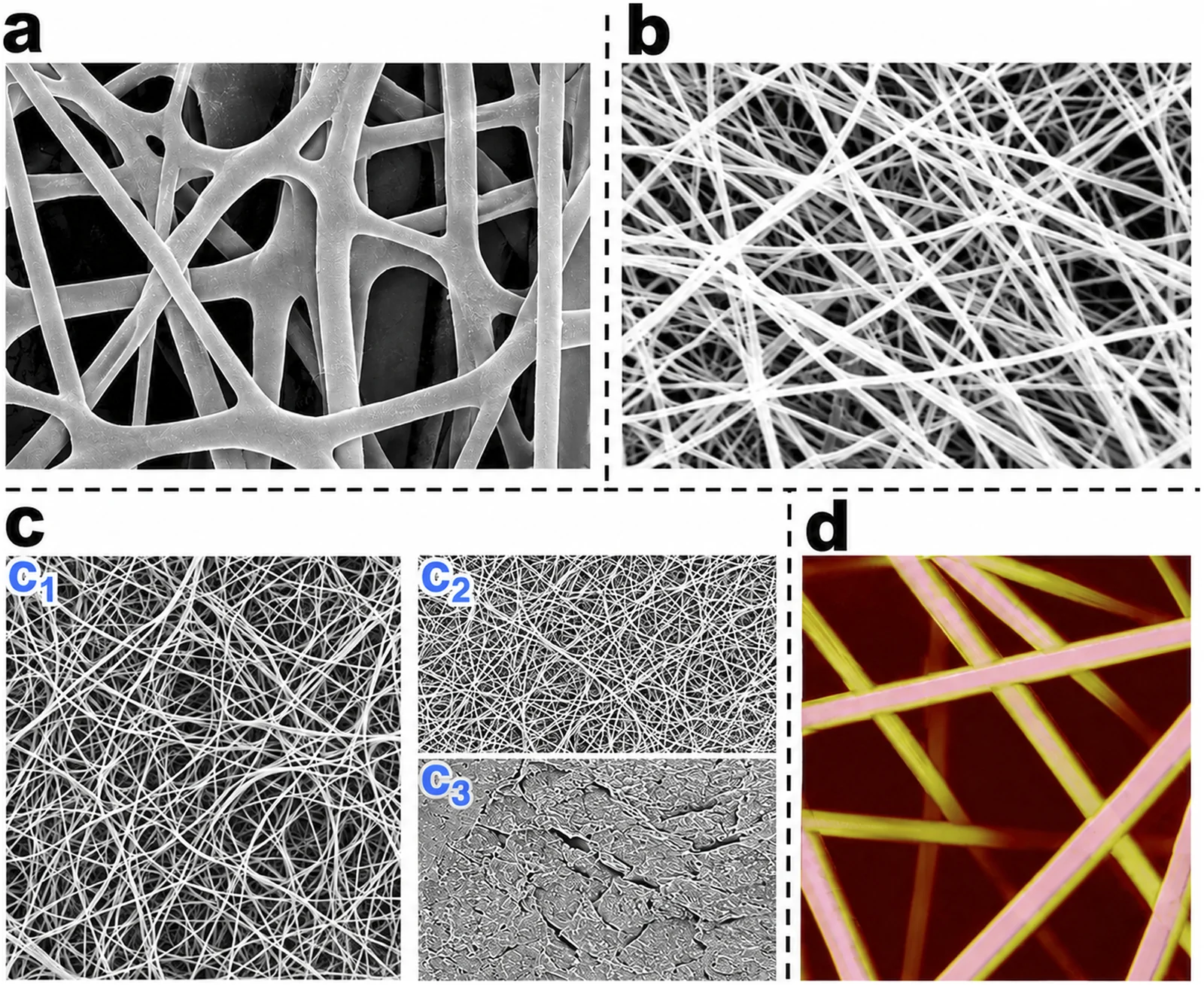
Representative SEM images illustrating the nanoscale morphology of FD buccal/oral NF systems. (a) PVA-based domperidone-loaded NFs exhibiting uniform, bead-free morphology with fiber diameters of 280–565 nm and a highly porous structure.^72^ (b) Pullulan/xanthan gum vitamin C-loaded NFs showing smooth, continuous fibers with average diameters of 181–261 nm and high nanoscale porosity.^77^ (c) Multilayer ciprofloxacin hydrochloride electrospun buccal patches revealing distinct layers (c_1_–c_3_) with an interconnected porous fibrous architecture that facilitates saliva infiltration.^78^ (d) AFM images of ondansetron-loaded PVA mats showing smooth low-roughness topography.^47^ The image fragments were adapted from different research papers: (a) adapted from ref. 72 with permission from Elsevier, copyright 2024; (b) adapted from ref. 77 under a Creative Commons Attribution 4.0 International License (CC BY), copyright 2024; (c) adapted from ref. 78 under a Creative Commons Attribution 4.0 International License (CC BY), copyright 2022; and (d) adapted from ref. 47 with permission from Elsevier, copyright 2016.

### 5.2 Solid-state characterization

DSC, XRD, and FTIR confirm amorphization (complete loss of crystalline melting and diffraction peaks) and drug–polymer interactions, which enhance solubility and dissolution in the buccal cavity.^80^ DSC of diosmetin membranes showed a drastic drop in fusion enthalpy (23.6 *vs.* 260.7 J g^−1^) (Fig. 5a), verifying amorphous dispersion;^81^ prochlorperazine NFs reduced crystallinity from 87% to 8% (Fig. 5b), accelerating release.^32^ Complete loss of XRD peaks in curcumin PVP NFs (Fig. 5c) and prochlorperazine-loaded fibers confirmed matrix embedding and enhanced permeation.^32,82^ FTIR of indomethacin-loaded cellulose mats showed hydrogen bonding without degradation (Fig. 5d), indicating compatibility.^80^ These techniques collectively demonstrate role of electrospinning in generating supersaturation, and thereby promoting the formation of amorphous solid dispersions.^79^

**Fig. 5:**
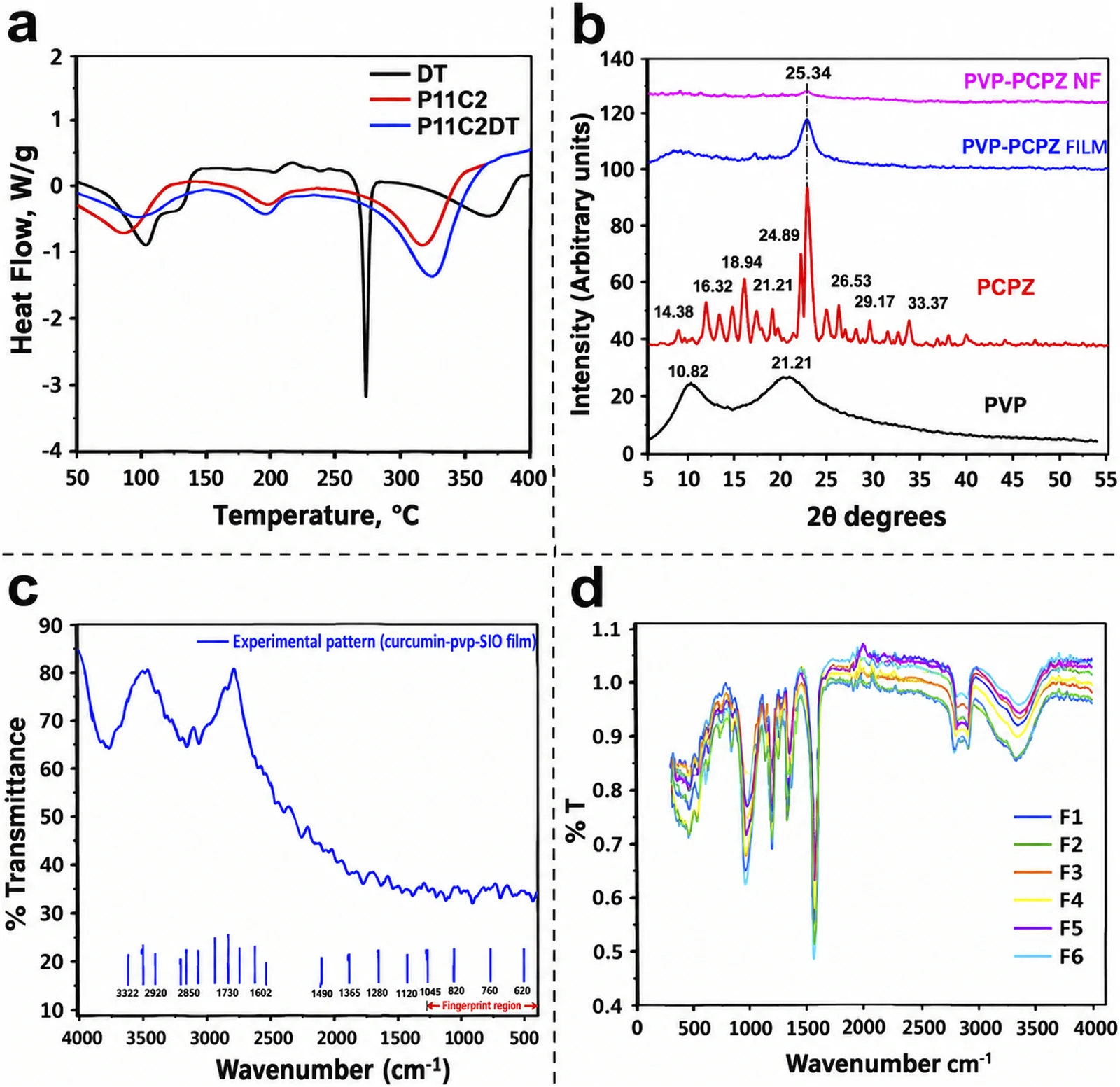
Representative solid-state characterization data confirming drug amorphization and drug–polymer compatibility in FD buccal NF systems. (a) DSC thermograms of diosmetin powder and a fibrous membrane showing drastic reduction in crystallinity and disappearance of the drug melting endotherm.^81^ (b) XRD patterns of pure prochlorperazine *versus* prochlorperazine-loaded NFs demonstrating complete loss of characteristic crystalline peaks of the drug after electrospinning.^32^ (c) XRD spectrum of curcumin-loaded PVP NFs, indicating the complete amorphous conversion of the drug in this form, which plays a huge role in improving the drug's solubility.^82^ (d) FTIR spectra of cellulose acetate, indomethacin, their physical mixture, and indomethacin-loaded buccal NF mats confirming successful drug–polymer interactions without chemical degradation.^80^ The image fragments were adapted from different research papers: (a) adapted from ref. 81 with permission from Wiley, copyright 2024; (b) adapted from ref. 32 with permission from Elsevier, copyright 2024; (c) adapted from ref. 82 with permission from Elsevier, copyright 2022; and (d) adapted from ref. 80 with permission from Elsevier, copyright 2017.

### 5.3 Hydration and disintegration assessment

Disintegration behavior represents the defining performance metric of FD buccal NF systems. Unlike conventional tablets, electrospun mats lack a pharmacopeial testing standard; therefore, modified *in vitro* methods are commonly employed to simulate saliva exposure. These include hydration tests, wet sponge or filter paper models, and small-volume simulated salivary fluid assays maintained at physiological temperature (37 °C).^68^ These methods typically involve placing a small section of the NF mat on a moistened substrate or adding microliter quantities of simulated saliva and visually timing the point of complete structural collapse or dissolution. High porosity drives capillary wicking, yielding 5–30 s disintegration without gel formation; low contact angles (<30°) predict spreading. For instance, Turanlı *et al.* achieved complete disintegration in as little as 1 s using small-volume simulated oral environment tests on PEO-based risperidone NFs (Fig. 6a).^83^ Alamer *et al.* reported 2 ± 1 s disintegration on moistened substrates for imeglimin-loaded buccal NFs (Fig. 6b).^31^ On the other hand, Abdelhakim *et al.* observed 0.5 s collapse in simulated saliva for Eudragit® E PO pediatric NFs.^84^ Bhardwaj *et al.* linked >80–95% porosity to instant wicking.^85^ These validate the design for rapid-onset therapy.

**Fig. 6:**
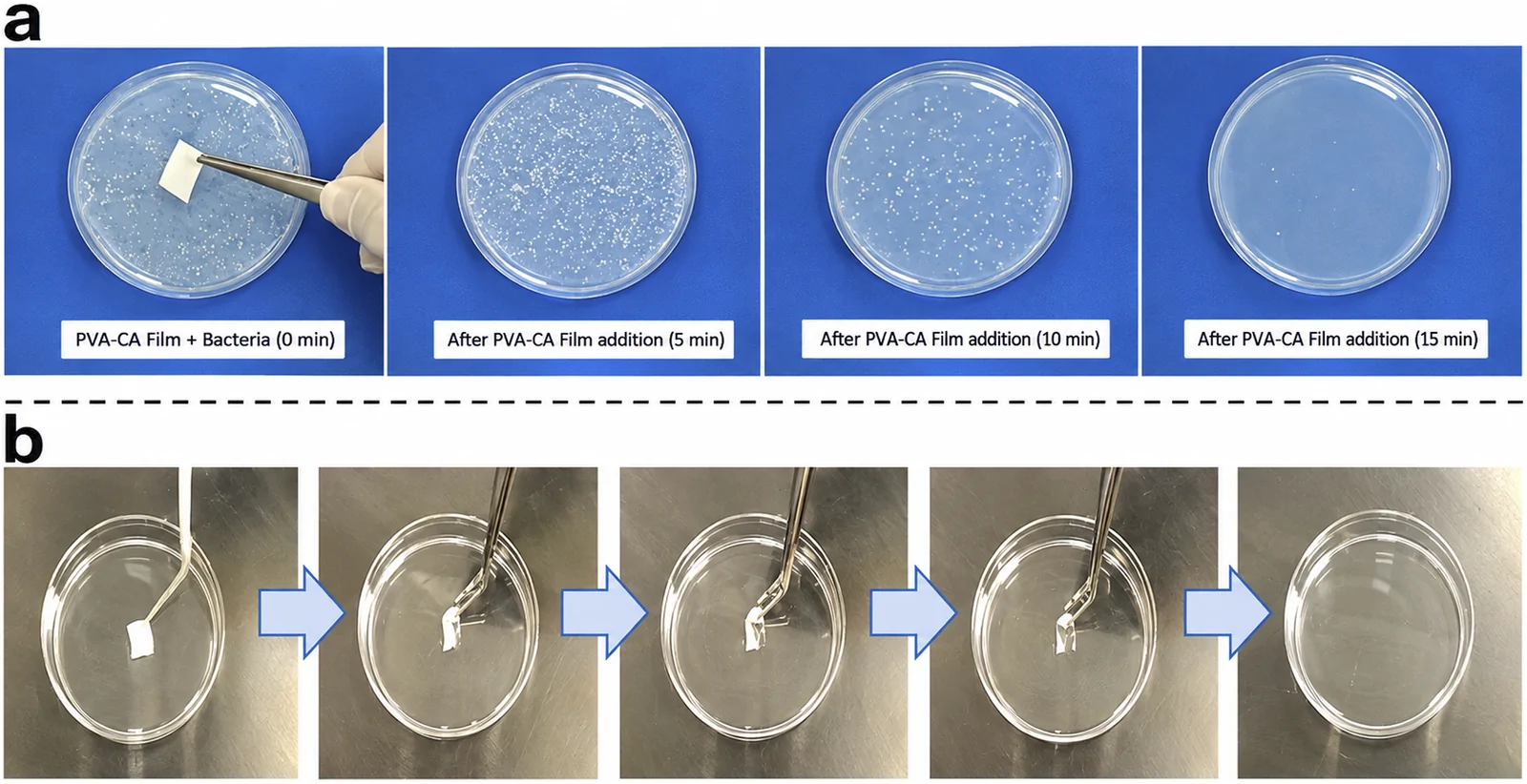
*In vitro* hydration and disintegration assessment of FD electrospun NF mats under buccal-mimicking conditions. (a) Time-lapse sequences of PEO-based risperidone NFs showing complete structural collapse within 1 second in a simulated oral environment.^83^ (b) Imeglimin-loaded buccal NFs on a Petri dish containing PBS (pH 6.8), demonstrating full disintegration in 2 ± 1 s at 37 °C.^31^ The image fragments were adapted from different research papers: (a) adapted from ref. 83 with permission from Elsevier, copyright 2024; and (b) adapted from ref. 31 under a Creative Commons Attribution 4.0 International License (CC BY), copyright 2023.

### 5.4 Mechanical and functional integrity

Although designed for rapid dissolution, FD buccal NF mats must possess sufficient mechanical integrity to withstand handling, packaging, and placement in the buccal cavity without tearing or crumbling. Key parameters commonly assessed include tensile strength, percentage elongation at break, Young's modulus, and folding endurance.^79^ A few of these properties are typically measured using universal tensile testing machines (stress–strain profiles at controlled crosshead speeds) and repeated manual or automated folding tests to simulate real-world manipulation.^75^ An optimal balance is essential: the mats must be flexible enough for conformal contact with the non-keratinized buccal mucosa (low Young's modulus and adequate elongation) while remaining robust enough to remain intact during administration (sufficient tensile strength and folding endurance). For instance, Asatiani *et al.* demonstrated that slight variations in HPMC/PEO-blend electrospun orodispersible films produced tensile strengths of 1.2–3.8 MPa (Fig. 7a and c), Young's moduli of 30–120 MPa (Fig. 7b), and elongation at break values of 8–25% (Fig. 7d), directly influencing handling robustness and fast oral disintegration performance.^86^ Łyszczarz *et al.* developed core–shell electrospun orodispersible films. They reported Young's modulus values of 11.96–16.81 N mm^−2^, tensile strength of around 0.20 N mm^−2^, and elongation at break <5%, confirming the mechanical robustness required for easy handling of fast-dissolving oral NF systems while enabling rapid disintegration in the oral cavity.^87^ These reconcile speed with practicality.

**Fig. 7:**
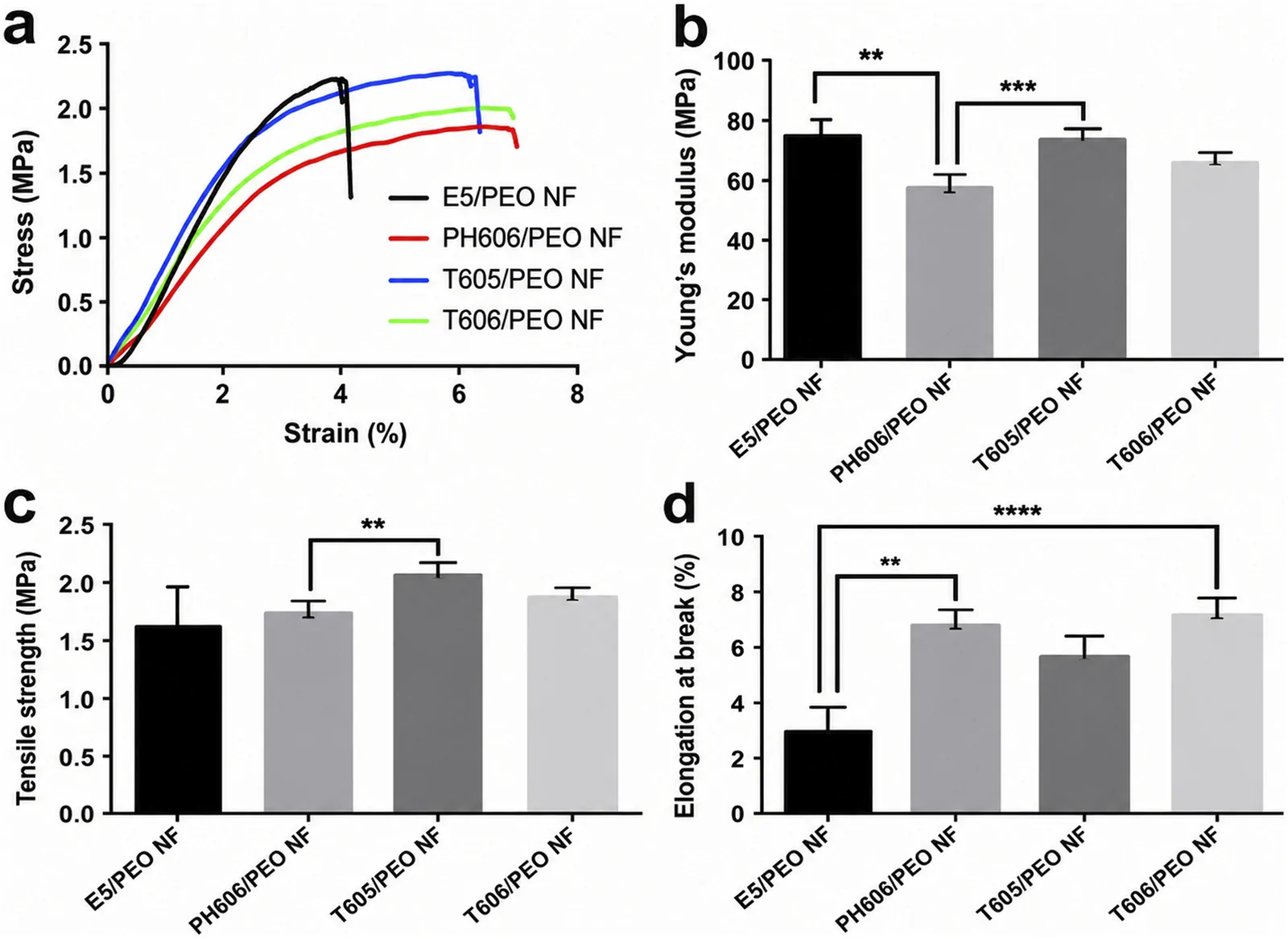
Mechanical characterization of FD orodispersible electrospun NF mats. (a and c) Representative stress–strain curves of HPMC/PEO-blend orodispersible NFs showing tensile strength (1.2–3.8 MPa), (b) Young's modulus (30–120 MPa), and (d) elongation at break (8–25%).^86^ The figure was adapted from ref. 86 under a Creative Commons Attribution 4.0 International License (CC BY), copyright 2023.

### 5.5 Dissolution and release kinetics

*In vitro* dissolution testing establishes the temporal profile of drug liberation from nanofibrous matrices. Due to the limited volume of saliva in the buccal cavity, modified dissolution setups using reduced media volumes are often employed to better approximate physiological conditions. Low-volume setups capture burst profiles (>80% release in <60 s from SA/*V* and amorphicity).^74^ Mathematical modeling using Higuchi or Korsmeyer–Peppas equations enables interpretation of diffusion contributions and polymer relaxation effects.^88^ Formulation-dependent modulation of release kinetics has been demonstrated in electrospun polysaccharide–PEO systems incorporating varying fractions of carboxymethylated curdlan. Submicron fibers with minimal polymer modification exhibited rapid initial drug liberation (>60% released within the first few minutes) (Fig. 8a), outperforming higher-modified formulations that shifted toward hydrogel-like controlled release. Kinetic analysis was well fit by the Peppas–Sahlin model (*R*^2^ > 0.99), indicating the presence of combined diffusion and polymer relaxation mechanisms.^89^ Németh *et al.* demonstrated polyaspartamide-based fast-dissolving nanofibrous matrices that released the incorporated model drug almost completely within 60 s (Fig. 8b) in simulated salivary fluid (pH 6.8).^90^ Thus, dissolution testing in these systems focuses on confirming immediate drug availability rather than sustained-release behavior.

**Fig. 8:**
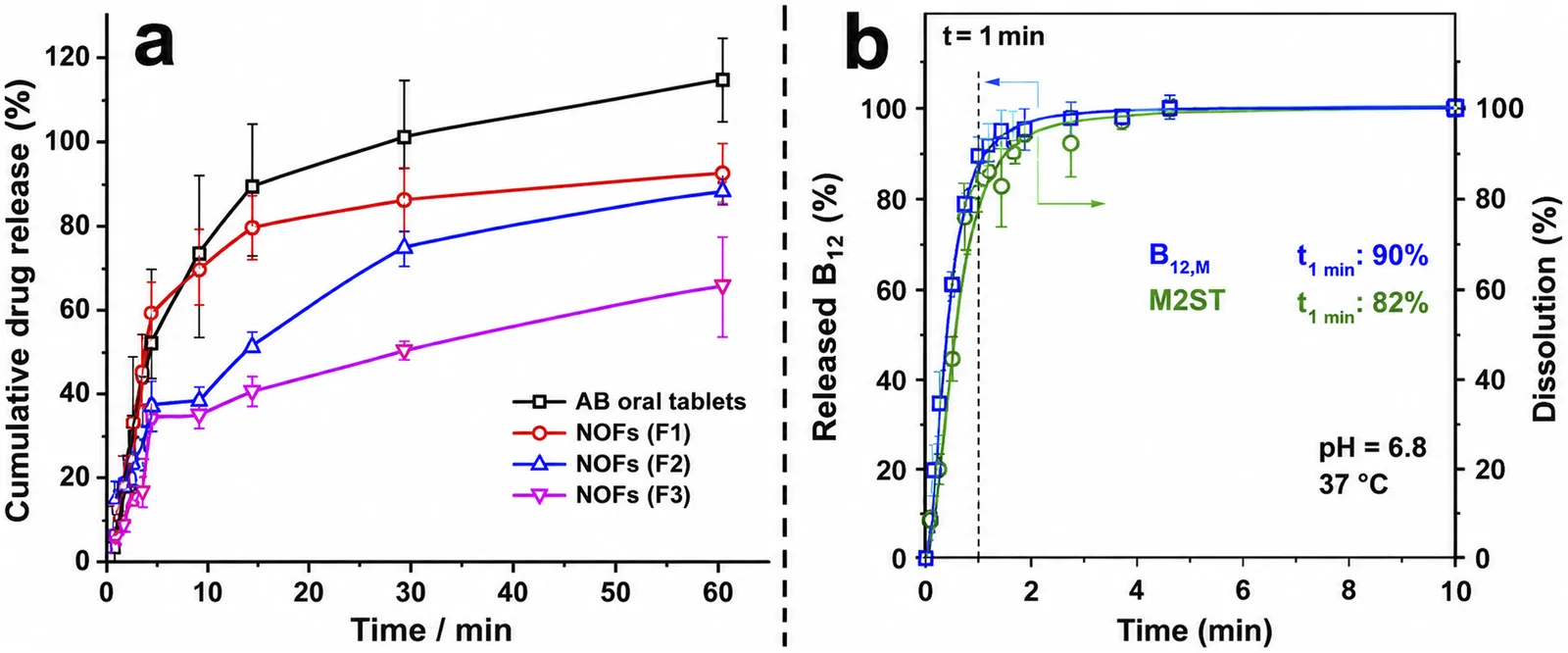
*In vitro* dissolution profiles illustrating early-time release behavior of fast-dissolving electrospun NF systems under simulated salivary/buccal conditions. (a) Cumulative drug release from carboxymethyl curdlan/PEO NFs: rapid burst (>60% within the first few minutes) at low modification *versus* slower, hydrogel-like release at higher modification.^89^ (b) Near-complete drug release within 60 s from polyaspartamide-based fast-dissolving nanofibrous matrices in simulated salivary fluid (pH 6.8).^90^ The image fragments were adapted from different research papers: (a) adapted from ref. 89 with permission from Elsevier, copyright 2022; and (b) adapted from ref. 90 with permission from Elsevier, copyright 2020.

### 5.6 Mucoadhesion and transmucosal evaluation

For buccal delivery applications, residence time and permeation potential are evaluated using texture analysis-based detachment testing and *ex vivo* mucosal models. Adhesion strength depends on polymer composition, surface roughness, and hydration behavior. This assessment typically involves applying a controlled force (*e.g.*, 3–5 g) to the NF mat against porcine buccal mucosa for a fixed contact time (30–60 s), followed by measurement of maximum detachment force and work of adhesion using a texture analyzer.^34,70^ Franz diffusion cell studies employing porcine buccal tissue are commonly used to quantify drug flux and assess permeation enhancement relative to conventional formulations by mounting the tissue in Franz diffusion cells containing simulated saliva in the donor compartment. These assessments confirm that rapid matrix disintegration does not preclude effective mucosal contact and drug transport.^91^ For instance, sublingual electrospun NFs of pramipexole exhibited reduced mucoadhesion (due to stronger polymer interactions) compared to cast films (Fig. 9a), providing sufficient transient attachment for initial placement without compromising the short mucosal exposure window of FD systems.^92^ In parallel, α-lactalbumin-based fast-dissolving NFs designed for rapid nicotine delivery in the oral cavity demonstrated significantly higher cumulative permeation across *ex vivo* porcine buccal mucosa compared to a nicotine solution control (Fig. 9b).^93^ These evaluations collectively confirm that rapid matrix disintegration does not preclude effective mucosal contact and drug transport while maintaining the transient interaction required for FD performance.

**Fig. 9:**
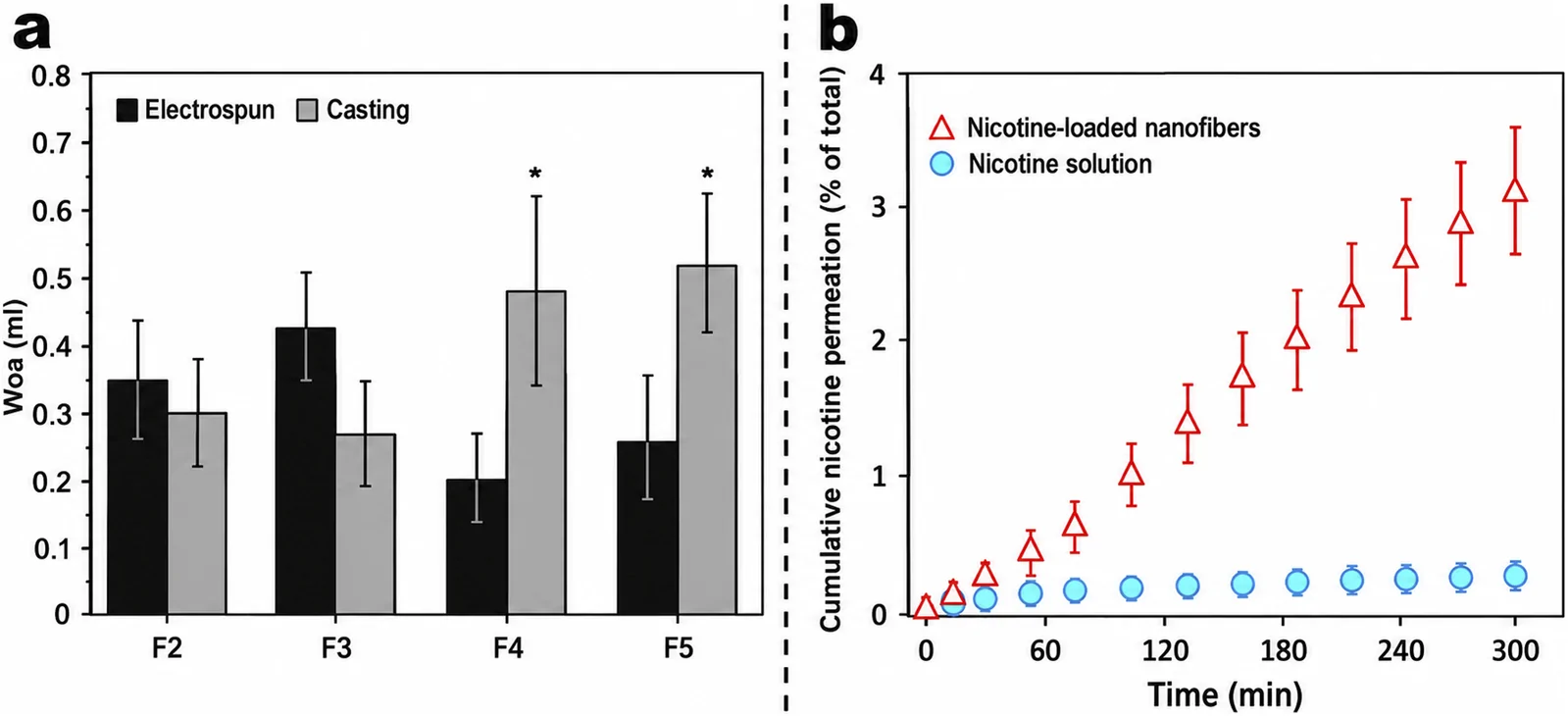
Mucoadhesion and transmucosal permeation evaluation of fast-dissolving electrospun NF mats for oral/sublingual delivery. (a) Texture analyzer mucoadhesion results (maximum detachment force and work of adhesion) for pramipexole-loaded sublingual electrospun NFs showing reduced adhesion, compared to cast films, suitable for short-exposure FD systems.^92^ (b) Cumulative nicotine permeation across *ex vivo* porcine buccal mucosa (Ussing chambers), demonstrating significantly higher flux from α-lactalbumin-based fast-dissolving NFs *versus* nicotine solution control.^93^ All tests used simulated salivary conditions to mimic the limited buccal/sublingual exposure window. The image fragments were adapted from different research papers: (a) adapted from ref. 92 with permission from Elsevier, copyright 2026; and (b) adapted from ref. 93 with permission from American Chemical Society, copyright 2020.

### 5.7 Stability considerations

Because electrospinning often produces amorphous solid-state fibers, stability evaluation under controlled temperature and humidity conditions is essential. ICH studies (40 °C/75% RH accelerated; 25 °C/60% RH long-term) track recrystallization (DSC/PXRD) and morphology shifts.^94^ Moisture uptake is particularly critical for hydrophilic polymers, as plasticization may alter mechanical properties or cause premature hydration.^95^ For instance, incorporation of hydroxypropyl-β-cyclodextrin into PVP-based NF mats loaded with meloxicam significantly enhanced long-term structural stability under ICH long-term conditions (25 °C/60% RH). The mats maintained smooth morphology and amorphous drug dispersion over six months of storage (Fig. 10a and b), with no recrystallization observed. Solid-state analyses (DSC and PXRD) confirmed preservation of the amorphous state, while rapid *in vitro* disintegration (∼14 s) remained unchanged.^96^ Similarly, escitalopram/quetiapine-loaded fast-dissolving PVP NFs retained their fully amorphous character and 2 s disintegration time after 4 months under ambient conditions, with no recrystallization observed by DSC and PXRD (Fig. 10c).^97^

**Fig. 10:**
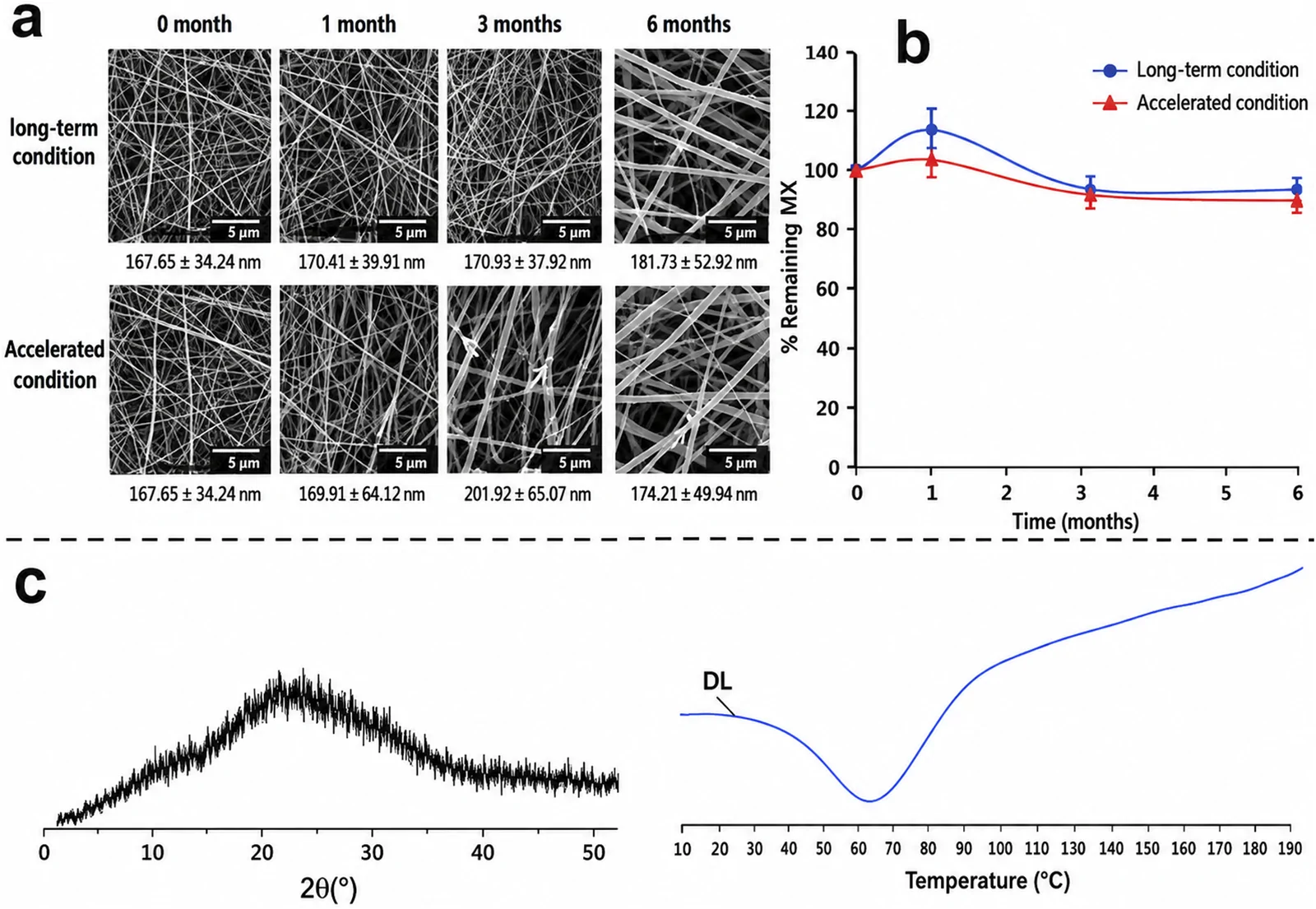
Long-term stability data of meloxicam-loaded PVP/HP-β-CD electrospun NF mats under ICH long-term storage conditions (25 °C/60% RH). (a) SEM images showing preserved smooth fiber morphology with no crystals after 0, 1, 3, and 6 months, (b) percentage of remaining meloxicam content under long-term (blue) *versus* accelerated conditions (red), demonstrating excellent chemical stability under relevant shelf-life conditions.^96^ (c) DSC and PXRD of escitalopram/quetiapine-loaded fast-dissolving NFs after 4 months of ambient storage (20–25 °C and 30–45% relative humidity), showing a broad halo and lack of drug endothermic peaks, respectively, confirming complete amorphicity and 2 s disintegration.^97^ This study followed ICH long-term storage conditions. The image fragments were adapted from different research papers: (a) and (b) adapted from ref. 96 with permission from Elsevier, copyright 2015; and (c) adapted from ref. 97 under a Creative Commons Attribution 4.0 International License (CC BY), copyright 2021.

Although the above studies demonstrate physicochemical stability under controlled storage conditions, successful clinical translation also requires consideration of the entire product lifecycle, including packaging, transportation, storage, and post-manufacturing quality assurance. Owing to their high surface area, porous architecture, and frequent use of hygroscopic polymers, electrospun buccal nanofibers are particularly susceptible to moisture uptake during storage and distribution.^98,99^ Consequently, high-barrier packaging systems such as aluminium–aluminium blister packs, multilayer foil sachets, or moisture-impermeable laminate pouches, often used in combination with desiccants, are likely to play a critical role in preserving the amorphous state and mechanical integrity of these dosage forms.^100^ In addition to routine physicochemical evaluation, lifecycle stability assessment should include periodic monitoring of residual solvent content in accordance with ICH Q3C guidelines, together with moisture content, drug-content uniformity, disintegration time, and dissolution performance throughout the proposed shelf life to ensure consistent product quality and therapeutic performance.^101^ Furthermore, transportation and storage under fluctuating temperature and humidity conditions should be considered during product development to ensure that formulation performance remains consistent under real-world distribution scenarios. Integrating these lifecycle considerations into stability protocols and product development strategies will facilitate regulatory evaluation, support scientifically justified shelf-life assignment, and accelerate the clinical and commercial translation of electrospun buccal nanofibers.

### 5.8 Current limitations of *in vitro* evaluation and future directions for pharmacopeial standardization

Despite extensive progress in the development of electrospun buccal nanofibers, their *in vitro* characterization remains constrained by the absence of standardized evaluation methodologies.^69^ Most current evaluation techniques have been adapted from conventional oral or transdermal dosage forms rather than being specifically developed for rapidly disintegrating electrospun buccal systems.^102^ Consequently, variations in artificial saliva composition, dissolution medium volume, mucosal substrates, residence time, and sampling conditions frequently result in inconsistent performance data across the literature.^69^ Among these methods, dissolution and permeation testing present the greatest translational challenge. Conventional USP dissolution apparatus generally operates under sink conditions using relatively large medium volumes, which poorly represent the buccal cavity where only a thin and continuously replenished saliva layer is available.^103^ Likewise, static Franz diffusion cells remain the most widely employed technique for evaluating buccal permeation because of their simplicity and reproducibility; however, they fail to reproduce continuous salivary turnover, oral movements, and the dynamic hydration–erosion processes that govern drug release from electrospun nanofibers *in vivo*.^104^ These physiological discrepancies limit the predictive capability of conventional *in vitro* assays despite their widespread acceptance in formulation development.

Recent advances in microfluidic technologies provide promising alternatives capable of bridging this gap between laboratory testing and physiological performance. Millifluidic and meso-fluidic dissolution–permeation platforms have been developed to operate under controlled flow conditions with low-to-medium volumes, and thereby provide better simulating salivary secretion while enabling simultaneous evaluation of dissolution and membrane permeation. Integration of biomimetic membranes with flow-based microfluidic systems further enhances physiological relevance by providing continuous acceptor-side perfusion and improved prediction of *in vivo* drug absorption.^105,106^ Looking ahead, greater emphasis should be placed on establishing harmonized testing methodologies specifically tailored for electrospun buccal dosage forms. Standardization of artificial saliva composition, dissolution volume, flow rate, membrane selection, mucoadhesion testing protocols, and data reporting would substantially improve reproducibility and facilitate interlaboratory comparison. Such efforts could ultimately support the development of pharmacopeial guidance for electrospun buccal formulations, thereby accelerating their regulatory evaluation and clinical translation.^69^ To facilitate direct comparison of the available characterization approaches, Table 2 summarizes the principal *in vitro* evaluation methods, their current applications, major limitations, and future opportunities for pharmacopeial standardization.

**Table 2:** Comparative overview of current *in vitro* evaluation methods for fast-disintegrating electrospun buccal nanofibers and future opportunities for standardization

*In vitro* evaluation method	Current applications	Major limitations	Potential standardization strategy	References
Disintegration testing	Determines film disintegration time	Lack of standardized apparatus specifically designed for electrospun buccal nanofibers; limited simulation of saliva volume and oral movements	Development of physiologically relevant low-volume dynamic disintegration systems	107
Swelling studies	Evaluates hydration and matrix expansion	Static immersion conditions poorly mimic intermittent saliva exposure	Dynamic saliva-flow platforms with controlled hydration	69
Mucoadhesion testing	Measures adhesion strength and residence time	Large variability in mucosal substrates, test conditions and reporting methods limits comparison between studies	Standardized artificial oral mucosa and harmonized testing protocols	107
Dissolution testing	Drug release evaluation	USP apparatus and large dissolution volumes do not represent buccal physiology	Millifluidic/microfluidic flow-through systems operating under physiological salivary flow	108
Franz diffusion/permeation	Drug permeation across biological membranes	Static donor/acceptor compartments cannot reproduce continuous saliva renewal or oral biomechanics	Flow-based microfluidic dissolution–permeation platforms and biomimetic membrane systems	108
Stability evaluation	Evaluates physical and chemical stability	No standardized protocol specifically addressing amorphous electrospun buccal products	Harmonized stability guidelines incorporating moisture uptake, residual solvent monitoring and amorphous-state assessment	10

## 6. Drug selection criteria for FD buccal NF mats

FD buccal NF mats excel for low-to-moderate-dose drugs (≤50 mg) requiring rapid systemic onset, where their burst release and amorphous dispersion address pharmacokinetic hurdles like FPM, poor solubility, or delayed absorption.^109^ Selection prioritizes molecules amenable to amorphization with moderate lipophilicity (log *P* ∼ 1–4 for epithelial flux), stability in saliva, and acute therapeutic needs, while avoiding high-dose compounds or those requiring prolonged residence that would overload the fibers or exceed the brief mucosal exposure window.^110^ Biologics and peptides are generally unsuitable due to enzymatic degradation in saliva, making small-molecule APIs that readily form amorphous solid dispersions the preferred candidates.^111^ This alignment maximizes the core advantages of FD systems: <30 s disintegration and ≤60 s release, which can improve bioavailability 2–3 times compared with conventional oral forms for FPM-prone drugs.^109^ Representative applications include antiemetics, analgesics, anxiolytics and antidiabetics, as summarized in Table 3. These examples illustrate how NF mats mitigate specific limitations: *e.g.*, ondansetron's extensive FPM (bioavailability < 60%) is bypassed *via* sublingual burst release, yielding >90% release in 90 s and faster *T*_max_;^47^ diclofenac's low solubility (log *P* = 4.5) benefits from PVP-induced amorphization, achieving 0.4 s disintegration and 3.7-fold higher *ex vivo* permeation.^25^ Pediatric-friendly risperidone NFs (1 s collapse) enable rapid antipsychotic onset without swallowing.^83^ Limitations include loading capacity (10–30 wt% to avoid beading) and potential local irritation at high concentrations, which redirects very high-dose drugs (*e.g.*, >100 mg paracetamol) to GI routes.^110^

**Table 3:** Representative drugs successfully incorporated into FD electrospun NF mats, illustrating how key pharmacokinetic and formulation limitations were addressed

Criteria/therapeutic need	Key features addressed	Representative drugs & FD NF performance	Log *P* range	Reference(s)
Low-dose, potent APIs (≤10 mg; high potency minimizes loading)	Rapid flux for precise dosing; suits pediatrics/geriatrics	Nicotine (sublingual/oral transmucosal): <60 s oral release, 3 times higher mucosal permeation *vs.* solution. Risperidone (oral/orodispersible): ∼1 s disintegration, full release <60 s for antipsychotic onset. Prochlorperazine (oral/orodispersible): <1 s collapse, 91% release in 2 min (antiemetic). Domperidone (oral/orodispersible): 4 s disintegration, 93% in 10 min	1–3	32, 72, 83 and 93
Extensive FPM (bioavailability <50%; hepatic degradation)	Direct systemic bypass; burst elevates *C*_max_	Ondansetron (sublingual): <10 s disintegration, >90% in 90 s; 2 times faster *T*_max_. Nifedipine/atorvastatin (oral/orodispersible): >85% in 10 min (coaxial core–shell). Glibenclamide (buccal): buccal NFs evade FPM, stable amorphous state	2–4	26, 47 and 112
Rapid onset required (acute conditions; *T*_max_ < 15 min needed)	Immediate hydration/supersaturation for flux	Sumatriptan: migraine relief NFs, <30 s dissolution. Prochlorperazine (oral/orodispersible): <1 s disintegration, antiemetic. Midazolam (oral/orodispersible): fast oral NFs for anxiolysis. Risperidone (oral/orodispersible): ∼1 s onset for acute psychosis	1.5–3.5	32, 83, 113 and 114
Moderate lipophilicity/permeability (log *P* ≈ 1–4; avoids extremes)	Balances solubility/flux; amorphization aids	Diclofenac sodium (buccal): 0.4 s disintegration, high *ex vivo* flux. Meloxica [*m*: ∼14 s, taste-masked *via* cyclodextrin. Piroxicam (sublingual): sublingual NFs, rapid permeation. Ibuprofen (oral/orodispersible): <30 s oral release, 95% dissolution	3–4.5	25, 49, 54 and 115
Salivary stability (resists dilution/enzymatic loss)	Transient supersaturation before washout	Meloxicam: amorphous stable 6 months, <14 s. Glibenclamide (buccal): saliva-stable buccal NFs. Paracetamol/caffeine (oral/orodispersible): fast oral NFs, no degradation. Levosalbutamol sulfate (sublingual): fast sublingual, intact release	0.5–2.5	49, 112, 116 and 117

## 7. Design considerations and translational barriers in FD buccal NF systems

Despite their advantages of instantaneous hydration and burst release within ∼60 s, the high porosity (>80%) and hydrophilic composition of FD electrospun buccal NF mats introduce inherent challenges in mechanical robustness, mucosal retention, solid-state stability, and scalability. These challenges, rooted in the very features that enable fast performance, can be addressed through targeted polymer blending, excipient optimization, process engineering, and quality-by-design (QbD) approaches, paving the way for GMP-compliant products.^118^

### 7.1 Design optimization under practical constraints

The nanoscale fibrous network that delivers rapid dissolution simultaneously results in low tensile strength (<2 MPa) and brittleness, making the mats prone to tearing during handling or placement. High drug loadings (>15 wt%) further disrupt fiber continuity and exacerbate fragility.^35,86,109^ Blending with reinforcing polymers such as pullulan or α-chitin increases toughness (1.5–3-fold improvement in modulus)^119,120^ while preserving <10 s disintegration, as shown in PVA-pullulan ondansetron mats that maintained 1.2 MPa tensile strength.^47^ A second challenge is the retention–dissolution paradox: although rapid collapse (<30 s) maximizes burst flux, it risks premature salivary washout (0.3–0.5 mL min^−1^ unstimulated flow), potentially reducing effective absorption.^24,34,83^ Transient mucoadhesive polymers, such as low-degree-of-substitution thiolated chitosan or chitosan-pullulan blends, extend residence time (work of adhesion ∼1 mJ cm^−2^) without forming viscous gels, thereby increasing flux 1.8-fold in porcine buccal models while still achieving <60 s release.^50,70^ Solid-state stability remains a major translational constraint because moisture-induced plasticization and recrystallization can compromise the rapid-dissolution advantage of amorphous nanofibers, underscoring the importance of moisture-resistant formulations and packaging strategies discussed in Section 5.7. From a formulation-design perspective, this constraint can be addressed through the incorporation of stabilizing excipients, such as hydroxypropyl-β-cyclodextrin, or by introducing hydrophobic components that reduce moisture-induced molecular mobility and inhibit recrystallization. Such approaches have been shown to preserve the amorphous drug state and maintain rapid disintegration and morphological integrity during storage.^49,121^ These excipient strategies reconcile speed with practical handling, retention, and shelf-life without compromising the thermodynamic advantages of FD mats.

### 7.2 Overcoming manufacturing and regulatory hurdles

Although laboratory-scale electrospinning has consistently demonstrated the feasibility of producing fast-disintegrating buccal nanofibers with excellent physicochemical characteristics, their translation to industrial manufacturing remains challenging.^37^ Conventional single-needle electrospinning is inherently limited by low production throughput (0.01–2 g h^−1^ per needle)^37,122^ and high sensitivity to process parameters (voltage, relative humidity, flow rate), which influence jet stability, fiber diameter, porosity, drug loading and residual solvent content.^123^ Consequently, these inconsistencies can significantly influence critical quality attributes (CQAs), including disintegration time, dissolution behaviour, dose uniformity, and batch-to-batch reproducibility, thereby complicating large-scale pharmaceutical manufacturing. To address these limitations, considerable efforts have focused on high-throughput electrospinning technologies, particularly multi-nozzle and needleless systems. Platforms such as NanoSpider™^124^ have increased production rates to approximately 100 g h^−1^ while maintaining sub-500 nm fiber diameters, rapid disintegration and burst drug release (<60 s), with reported batch-to-batch variability below 5% in scaled PVP–ketoprofen formulations.^35,125^ Nevertheless, maintaining a uniform electric field, stable polymer flow, and consistent fiber morphology across multiple spinning jets remains a significant engineering challenge that requires further optimization before routine pharmaceutical manufacturing can be achieved.

Another important consideration during scale-up is residual solvent control. Organic solvents frequently employed during electrospinning, particularly dimethylformamide (DMF), may raise concerns regarding mucosal safety, residual solvent limits, and regulatory compliance. Consequently, increasing attention has shifted toward aqueous electrospinning systems based on water-soluble polymers such as poly(vinyl alcohol) (PVA) and pullulan, which have been shown to reduce residual solvent levels by more than 90% while maintaining rapid disintegration and burst drug-release characteristics consistent with International Council for Harmonisation (ICH) guidelines.^118,126^

Manufacturing robustness can be further enhanced through the implementation of QbD principles combined with Design of Experiments (DOE) and Process Analytical Technology (PAT). Systematic optimization has demonstrated improved control and prediction of critical quality attributes, including fiber morphology, permeation behaviour, and process-related performance, when critical process parameters are systematically optimized using QbD, DOE, and data-driven modelling approaches.^127,128^ In parallel, PAT enables real-time monitoring of solution viscosity, electrical conductivity, environmental conditions, jet stability, and residual solvent content, thereby improving process control, product consistency, and manufacturing reliability. Collectively, these approaches support regulatory Chemistry, Manufacturing, and Controls (CMC) expectations by strengthening process understanding, ensuring robust control of CQAs, and facilitating consistent product quality throughout manufacturing.

From a regulatory perspective, electrospun buccal NF mats continue to present classification and quality-assessment challenges because specific regulatory requirements for electrospun nanofiber-based buccal systems remain insufficiently harmonized. Current regulatory frameworks generally evaluate buccal and orodispersible dosage forms according to their composition, route of administration, manufacturing process, and intended therapeutic function; however, specific guidance addressing the nanoscale fibrous architecture, critical quality attributes, and manufacturing controls of electrospun buccal NF mats remains limited.^129,130^ Concurrently, the development of harmonized pharmacopeial standards addressing nanofiber-specific quality attributes such as disintegration performance, mechanical integrity, humidity stability, and residual solvent specifications is expected to accelerate commercialization further. Together with continued advances in sustainable solvent systems and scalable electrospinning technologies, these developments are likely to facilitate the successful industrial translation of electrospun FD buccal nanofiber drug-delivery systems.^98,131^

Recent advances have also focused on hybrid fabrication technologies that combine electrospinning with complementary fabrication methods, including three-dimensional (3D) printing and continuous production platforms. These integrated approaches enable precise control of multilayer architectures, improve dose uniformity, and facilitate scalable production while preserving the unique physicochemical advantages of electrospun nanofibers.^132^ In particular, layer-by-layer electrospinning-3D printing systems have demonstrated enhanced structural reproducibility and manufacturing flexibility, making them promising candidates for personalized drug-delivery systems and industrial translation.^133^ Furthermore, the transition from conventional single-nozzle electrospinning to multi-nozzle and needleless manufacturing platforms has substantially improved production throughput and batch-to-batch consistency.^134^ Beyond aqueous electrospinning, solvent-free melt electrospinning has emerged as a promising alternative for minimizing residual solvent concerns and simplifying regulatory compliance. However, further optimization is required for broader pharmaceutical applications.^135^ Collectively, these advances provide a framework for improving manufacturing consistency, controlling CQAs, and strengthening the quality and regulatory readiness of electrospun buccal nanofiber products.

### 7.3 Addressing clinical and patient-centric realities

*In vivo* performance must contend with salivary variability (0.5–1.5 L per day plus speech-induced shear),^136^ which can compress the absorption window and cause 30–50% dose loss in non-adhesive mats unless permeation kinetics outpace dilution.^24^ Optimized supersaturation-driven flux (per Fick's law) mitigates this, as evidenced by α-lactalbumin nicotine NFs that achieved 2-fold higher bioavailability despite salivary flow.^93^ Clinical evidence is still emerging. Most data remain *in vitro* or *ex vivo*, but early human studies on related fast-dissolving oromucosal platforms are promising; for example, a phase I psychophysiological study of caffeine-loaded oral films demonstrated detectable neuromodulatory effects,^137^ while preclinical electrospun caffeine NFs consistently disintegrate rapidly.^116^ A 2025 preclinical study on sublingual pramipexole NFs for relief of Parkinson's dyskinesia further supports the potential for rapid onset.^92^ Preclinical animal data from our own laboratory further support the translational potential of these systems. For instance, Shafi *et al.* developed a super-disintegrating nanofiber patch of zolmitriptan using a polyvinylpyrrolidone matrix and quality-by-design optimization. The patch disintegrates in <30 s and releases ∼82% of the drug within 1 min *in vitro*. *Ex vivo* studies demonstrated high flux across porcine buccal mucosa, while *in vivo* rat pharmacokinetic experiments demonstrated faster *T*_max_, higher *C*_max_, and significantly improved brain-to-plasma ratios compared with conventional oral tablets. These results confirm that FD nanofiber patches can achieve rapid systemic absorption and efficient brain targeting for acute migraine relief.^138^ Thus, FD NF mats represent a simple design optimized for onset speed, yet their translational success depends on balancing fragility, retention, stability, and scalability through the engineering solutions outlined above.

Despite the substantial growth in research on electrospun fast-disintegrating nanofibers, clinical translation remains at an early stage. Most published studies are limited to proof-of-concept formulation development, physicochemical characterization, and preclinical evaluation, whereas evidence from human clinical studies remains scarce.^139^ Furthermore, no electrospun fast-disintegrating buccal pharmaceutical product has yet received regulatory approval from major agencies such as the US Food and Drug Administration (FDA) or the European Medicines Agency (EMA).^140^ The current clinical translation landscape of electrospun fast-disintegrating buccal nanofibers is summarized in Table 4, highlighting the present level of evidence, major translational barriers, and future priorities for clinical implementation.

**Table 4:** Current clinical translation status of electrospun fast-disintegrating buccal nanofibers from laboratory research to commercialization

Translation stage	Current status	Representative examples	Major barriers	Key references
Proof-of-concept (*in vitro*)	Established	Numerous electrospun fast-disintegrating oral and buccal nanofiber formulations demonstrating rapid disintegration, amorphization, and enhanced dissolution	Limited standardization of characterization methods	30
Preclinical (*ex vivo*/*in vivo*)	Increasing evidence	Buccal, sublingual and oral mucosal nanofiber systems evaluated in animal and *ex vivo* permeation studies	Limited pharmacokinetic and long-term safety data	141
Clinical evaluation	Very limited	Human clinical evidence for electrospun fast-disintegrating buccal nanofibers remains extremely limited; available human data from related fast-dissolving oromucosal platforms provide supportive but indirect translational evidence	Lack of GMP-scale manufacturing, regulatory pathway, and standardized quality specifications	139
Commercial products	No approved electrospun fast-disintegrating buccal pharmaceutical product	No FDA- or EMA-approved fast-disintegrating buccal pharmaceutical product based specifically on electrospun nanofibers was identified in the literature and regulatory sources examined for this review	Scale-up, batch reproducibility, regulatory approval, pharmacopeial standards	139
Future translation	Highly promising	Continuous electrospinning, PAT, QbD, needleless electrospinning, green manufacturing	Clinical validation and regulatory harmonization	127

## 8. Conclusion and future directions

FD electrospun buccal NFs represent a promising platform for achieving rapid hydration, drug dissolution, and mucosal drug delivery within the limited exposure window of the oral cavity. Their high surface area, interconnected porous architecture, and ability to stabilize drugs in amorphous states provide important advantages over conventional buccal dosage forms, particularly for drugs requiring rapid onset of action or enhanced dissolution. However, successful development of these systems requires coordinated optimization of material properties, polymer–drug interactions, fiber architecture, and electrospinning parameters to achieve the desired balance between rapid disintegration, drug-loading capacity, mechanical integrity, mucosal retention, and solid-state stability. Despite substantial progress at the laboratory scale, important challenges remain before FD electrospun buccal nanofibers can achieve broader clinical and industrial translation. These include limited standardization of *in vitro* evaluation methods, moisture sensitivity and physical instability of amorphous drug states, residual solvent control, batch-to-batch reproducibility, scale-up of electrospinning processes, and the lack of harmonized regulatory and pharmacopeial requirements specific to nanofiber-based buccal dosage forms. Addressing these challenges will require greater integration of physiologically relevant testing platforms, lifecycle-oriented stability assessment, QbD, PAT, and scalable manufacturing approaches. The future development of FD electrospun buccal NFs is expected to extend beyond laboratory optimization towards digitally controlled and sustainable manufacturing platforms. Artificial intelligence (AI) and machine-learning (ML) algorithms are increasingly being explored to optimize electrospinning parameters, predict fiber morphology, and minimize experimental variability through data-driven process modelling.^142,143^ Future translation of electrospun buccal NFs is also expected to benefit from advances in hybrid manufacturing technologies, continuous pharmaceutical manufacturing, and environmentally sustainable processing strategies.^144^ Together with AI-assisted process optimization, real-time quality monitoring, and harmonized regulatory frameworks, these innovations are expected to improve manufacturing robustness, accelerate commercialization, and facilitate the widespread clinical adoption of personalized electrospun buccal drug-delivery systems.^98^ Ultimately, the successful translation of FD electrospun buccal nanofibers will depend on moving from formulation-centric optimization toward an integrated development strategy encompassing material selection, process control, physiologically relevant performance testing, stability, scalable manufacturing, and clinical validation. Continued collaboration among formulation scientists, process engineers, analytical researchers, clinicians, and regulatory agencies will be essential for establishing robust quality standards and demonstrating the clinical value of these systems. With these advances, electrospun buccal nanofibers have the potential to progress from promising laboratory-scale platforms to clinically relevant and commercially viable drug-delivery systems.

## Conflicts of interest

The authors declare no conflict of interest.

## Data Availability

No new data were created or analyzed in this study. Data sharing is not applicable to this article.
